# The ATPase activity of yeast chromosome axis protein Hop1 affects the frequency of meiotic crossovers

**DOI:** 10.1093/nar/gkae1264

**Published:** 2024-12-27

**Authors:** Kshitiza M Dhyani, Suman Dash, Sameer Joshi, Aditi Garg, Debnath Pal, Koodali T Nishant, Kalappa Muniyappa

**Affiliations:** Department of Biochemistry, Indian Institute of Science, CV Raman Road, Bengaluru 560012, India; School of Biology, Indian Institute of Science Education and Research, Maruthamala(PO), Vithura, Thiruvananthapuram 695551, India; School of Biology, Indian Institute of Science Education and Research, Maruthamala(PO), Vithura, Thiruvananthapuram 695551, India; Computational and Data Sciences, Indian Institute of Science, CV Raman Road, Bengaluru 560012, India; Computational and Data Sciences, Indian Institute of Science, CV Raman Road, Bengaluru 560012, India; School of Biology, Indian Institute of Science Education and Research, Maruthamala(PO), Vithura, Thiruvananthapuram 695551, India; Department of Biochemistry, Indian Institute of Science, CV Raman Road, Bengaluru 560012, India

## Abstract

*Saccharomyces cerevisiae* meiosis-specific Hop1, a structural constituent of the synaptonemal complex, also facilitates the formation of programmed DNA double-strand breaks and the pairing of homologous chromosomes. Here, we reveal a serendipitous discovery that Hop1 possesses robust DNA-independent ATPase activity, although it lacks recognizable sequence motifs required for ATP binding and hydrolysis. By leveraging molecular docking combined with molecular dynamics simulations and biochemical assays, we identified an ensemble of five amino acid residues in Hop1 that could potentially participate in ATP-binding and hydrolysis. Consistent with this premise, we found that Hop1 binds to ATP and that substitution of amino acid residues in the putative ATP-binding site significantly impaired its ATPase activity, suggesting that this activity is intrinsic to Hop1. Notably, K65A and N67Q substitutions in the Hop1 N-terminal HORMA domain synergistically abolished its ATPase activity, noticeably impaired its DNA-binding affinity and reduced its association with meiotic chromosomes, while enhancing the frequency of meiotic crossovers (COs). Overall, our study establishes Hop1 as a DNA-independent ATPase and reveals a potential biological function for its ATPase activity in the regulation of meiotic CO frequency.

## Graphical Abstract

**Graphical Abstract:**
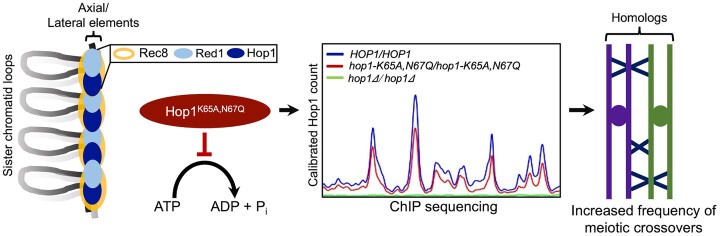

## Introduction

In almost all sexually reproducing organisms that have been studied so far, two prominent processes occur during meiotic prophase I; they include the assembly of the synaptonemal complex (hereafter SC), a zipper-like tripartite protein scaffold, which connects the axes of homologs along their lengths and the formation of crossovers (hereafter COs) between homologous chromosomes (1–6). Early electron microscopy studies demonstrated that the SC structure is evolutionarily conserved, typically comprises two lateral elements (hereafter LEs), separated by a central region, and consecutive arrays of sister chromatin loops tethered to the LEs (1,2). Despite the ultrastructural and functional conservation of SC across diverse eukaryotes, the proteins that make up the SC exhibit considerable sequence diversity (6–10). However, very little is known about the biochemical mechanisms underlying the function of SC components. Multiple studies have observed that failure to properly assemble the SC structure or malfunction of its components are often associated with chromosome aneuploidy, which is the major cause of infertility, miscarriages and congenital birth defects in humans (11–15). Therefore, isolation and biochemical characterization of the full repertoire of SC components may provide valuable insights into the molecular origin of such defects.

Broadly speaking, *Saccharomyces cerevisiae* has been widely used as a model organism for studying the SC components implicated in homolog juxtaposition during meiosis (2,5,16,17). In this system, previous studies have documented that the major components of the meiotic chromosome axes, also called LEs, include a meiosis-specific, DNA-binding Rec8-cohesin and two ‘core’ proteins: Red1 and the HORMA-domain containing Hop1 (18–22). Of these proteins, Hop1 is the most studied both *in vitro* and *in vivo*. Along with its binding partner Red1, Hop1 has been previously shown to localize throughout the length of chromosome axes, exhibiting a punctate staining pattern during interphase and early prophase in a manner dependent on Rec8-cohesin (21,23,24); however, in the absence of Rec8, Hop1 becomes essential for recruiting Red1 to the chromosome axis (25,26). These findings support the view that binary interaction between Red1 and Hop1 is important for their loading onto the chromosomal axis sites. Aside from its structural role as a component of SC, Hop1 has been implicated in various meiosis-based processes, including prophase checkpoint activation, formation of programmed double-strand breaks (hereafter DSBs) and repair, and homolog coalignment/synapsis (25,27–29), but our understanding of how Hop1 prioritizes these meiosis-specific processes and the mechanism(s) that regulate its function is limited.

The *S. cerevisiae* checkpoint activating protein Pch2, which has been previously identified as a AAA+ ATPase, inhibits Tel1 and Mec1-mediated Hop1 phosphorylation (30) and then dislodges the latter from the synapsing axes, thereby limiting DSB formation (23,24,31–33). Analogously, the Red1-dependent Hop1 phosphorylation by Tel1/Mec1 kinases triggers DNA repair via an inter-homologue dependent pathway (28,34–36). Paradoxically, Pch2 together with Hop1 positively regulates the meiotic recombination checkpoint response (37,38). Additional studies have demonstrated that overexpression of Red1 suppresses or enhances certain non-null *hop1* mutants (39,40). Likewise, Hop1 overproduction rescues the meiotic defects exhibited by *red1-K348E* mutant (41). On the other hand, co-overexpression of Hop1 with Red1 failed to rescue the *zip1* mutation, whose wild-type (WT) allele encodes a component of the SC transverse filament (42). Studies have shown that genetic disruption of *RED1* or *HOP1* caused cells to display reduced levels of Spo11-induced DSBs to 10–30% relative to the WT cells (25,27,41,43–45).

Emerging evidence indicates that the Hop1 orthologues exist in many eukaryotic species, including *Schizosaccharomyces pombe* (46), *Sordaria macrospora* (47), *Vanderwaltozyma polyspora* (48), *Arabidopsis thaliana* (49), *Caenorhabditis elegans* (50,51) and mammals (52–54), among others. Based on primary sequence similarity, a multifunctional protein–protein interaction module termed the HORMA domain (for Hop1, Rev7 and Mad2) was first identified in *S. cerevisiae* Hop1, Rev7 and Mad2 proteins (55) and subsequently found in both pro- and eukaryotes in a diverse range of proteins (56–58). It has been previously demonstrated that *S. cerevisiae* Hop1 exists in two forms, ‘inactive’ and ‘active’, and that Pch2 facilitates the transition from the ‘inactive’ to the ‘active’ configuration by releasing the safety belt lock from the HORMA domain (59,60). We found evidence that Hop1 contains two distinct regions: a protease-sensitive N-terminal HORMA region and protease-resistant C-terminal domain (hereafter Hop1-CTD) harboring the Cys_2_/Cys_2_ zinc finger motif (61). Our further studies showed that Hop1 folds GC-rich single-stranded DNA (ssDNA) into G4 DNA, binds more robustly to G-quadruplex DNA and to the Holliday junction (hereafter HJ) than double-stranded DNA (dsDNA) and ssDNA (61–64) via a central Cys_2_/Cys_2_ zinc finger motif (64–66). These findings are consistent with a recent study reporting that the *V. polyspora* Hop1 central region, comprising residues 317–535, binds to bent nucleosomal DNA (48), reinforcing the notion that Hop1 has a higher affinity to altered DNA structures (63,64).

By leveraging various experimental approaches, we previously demonstrated that Hop1 possess the unique ability to promote pairing between duplex DNA molecules involving GC-rich regions and that Red1, which lacks DNA/DNA pairing activity, potentiates its DNA pairing activity (66–70). Intriguingly, in line with these observations, atomic force microscopy imaging showed that Hop1 and Hop1-CTD independently promote pairing between duplex substrates and inter- and intramolecular bridging of DNA segments (61,71). However, ectopic expression of Hop1-CTD failed to rescue the spore viability defects of the *hop1Δ/hop1Δ* strain (61). This puzzling result can be explained by considering that the N-terminal HORMA domain may be involved in the recruitment of partner proteins such as Red1 to the chromosome axis. Curiously, genome wide profiling data on Hop1/Red1 association with meiotic chromosomes is somewhat controversial: while a recent study found no enrichment of Hop1 and Red1 in G-quadruplex forming regions (72), work from the Kleckner lab has demonstrated a striking correlation between the binding of Red1 and GC-rich genomic regions (73,74). While the specific reason for this apparent discrepancy remains unclear, we believe that it may be due to different techniques used in these two studies.

In this work, we describe our discovery that Hop1 has DNA-independent ATPase activity, despite lacking known sequence motifs needed for ATP-binding and hydrolysis. Using a multipronged approach, including molecular docking, molecular dynamics (MD) simulations and biochemical assays, we identified an ensemble of five amino acid residues in Hop1 that could potentially participate in ATP binding and/or hydrolysis. To test the functionality of these residues, we created constructs with single and double amino acid substitutions in the ATP-binding site of Hop1. Notably, we found that K65A and N67Q substitutions in the Hop1 N-terminal HORMA domain synergistically abrogated Hop1’s ATPase activity, significantly impaired its affinity for the HJ and reduced its association with meiotic chromosomes, but reciprocally increased the frequency of COs during meiosis. These findings collectively suggest that Hop1 is a novel DNA-independent ATPase and reveal a potential biological function for its ATPase activity in the regulation of meiotic CO frequencies.

## Materials and methods

### Biochemicals, DNA oligonucleotides, antibodies, bacterial strains and plasmids

Fine analytical grade chemicals were purchased from Sigma-Aldrich (St. Louis, MO) or GE Healthcare Life Sciences (Piscataway, NJ). The restriction endonucleases, Phusion high-fidelity polymerase, DNA ligase, T4 polynucleotide kinase and dNTPs were obtained from either Sigma-Aldrich (St. Louis, MO, USA) or New England Biolabs (Ipswich, MA, USA). The [γ-^32^P]ATP was procured from either the Bhabha Atomic Research Centre (Mumbai, India) or Revvity, Inc. (Waltham, USA). The DNA oligonucleotides (ODNs), SYPRO Orange, 4′,6′-diamidino-2-phenylindole (DAPI), NTPs, yeast and bacterial culture media components, and antibiotics were purchased from Sigma-Aldrich (St. Louis, MO, USA). The Ni^2+^-NTA resin, Sepharose G-50, isopropyl-β-D-thiogalactopyranoside (IPTG) and molecular weight standards were purchased from G-Biosciences (St. Louis, MO, USA). The ODNs containing 6-carboxyfluorescein (6-FAM) at the 5′-end were obtained from Genei Laboratories Pvt. Ltd. (Bengaluru, India). The Monolith NT.115 series Microscale Thermophoresis (MST) capillaries were obtained from Biobeams Scientific Instruments (Coimbatore, India). The rabbit reticulocyte lysate kit was procured from the Promega Corporation (New Delhi, India). The *Escherichia coli* strains and pET-28a (+) plasmid vector were obtained from Novagen (Madison, USA). The plasmids pFA6a-hphNT1 and pFA6a-kanMX4 were obtained from EUROSCARF (Oberursel, Germany). The ChIP grade anti-Hop1 polyclonal antibodies were a kind gift from Michael Lichten, NIH, Bethesda. The anti-His polyclonal antibodies and anti-rabbit antibodies were purchased from Santa Cruz Biotechnology (Texas, USA).

### Building the apo-Hop1 model

The protein sequences bearing homology to Hop1 were extracted from the MODBASE database (https://modbase.compbio.ucsf.edu/), using the BLAST program (https://blast.ncbi.nlm.nih.gov/) (75). The Hop1 modeling was guided by seven protein templates with *E*-values close to zero and negative zDOPE values. The structures were generated using MODELLER version 9.21 (76). The template information available for Hop1 spans the amino acid residues (aa) 9–277 and 423–584. The template corresponding to PDB ID: 3bar was used to guide the loop modeling. The segment from 278–422 did not yield any template from the Protein Data Bank (PDB). Therefore, a previously built model for the zinc finger motif (343–378) of Hop1 was used to guide the structure of this region (67). The PSIPRED program was used to predict elements of protein secondary structure in the regions spanning residues 278–342 and 379–422 (77). This analysis revealed that the regions spanning aa residues 279–342 and 299–309 form α-helix and 319–322, 329–333 and 336–337 aa regions fold into strands. Further, the residues 385–403 and 410–419 were found to form helical regions. The PSIPRED program could not predict a secondary structure for the residues in segments 278–342 and 379–422. To address this, we manually built coordinates for all the secondary structure in the 278–342 and 379–422 aa regions. The coordinates were provided to the Modeller software which generated the apo-Hop1 models using default parameters, of which the top five models were saved for analysis. The first round of model building was unsatisfactory in terms of structure compactness and deviant torsion parameters in some helical regions. Therefore, in the second round, several models were generated by deleting residues of varying lengths in the aa segment 278–422 and enforcing strict helical parameters. Of these, the model with deleted segments spanning 296–422 aa region was found to be reasonably compact. The rationale for following this approach was obtained from the initial model (first round), where the segment 278–422 was found juxtaposed in a groove created by the N- (1–277) and the C-terminal (423–605) segments.

We also built the N- and C-terminal segments individually to verify if the models built had significant differences compared with the initial model. In the third round, we included the coordinates of the model built in the second round as the template, the individual N- and C-terminal segments, the manually built secondary structures and the zinc finger to model the final structure. The top five models were saved and visually inspected in stereo for proper placement of the helix where the hydrophilic side should be solvent-exposed, and the hydrophobic side buried. We also analyzed the models based on the N- and C-termini proximities. A final mix-and-match of the terminal segments was performed to create the final structure with a DOPE value of -44590. This model did not have the ATP bound; therefore, we call it an apo-Hop1 model.

### Molecular docking and MD simulations

To identify the frames suitable for molecular docking of ATP docking, all-atom explicit solvent MD simulations were performed on the Hop1 structure, using the GROMACS software (78) and CHARMM27 (79) as a force field for 250 ns at 300 K temperature. The MD trajectory was recorded every 10 ps during the unconstrained dynamics simulation. The trajectory was visualized and two frames, at 108360 ps (frame 1) and 140310 ps (frame 2), were identified for docking. For both the frames, the ATP binding sites were determined using the results from Autodock Vina 1.1.2. Of the top 10 hits with the lowest energy score (−Δ*G*), the docked structure ranked 1 was assessed for stable *in silico* docking of ATP and all-atom MD simulations (200 ns) as described above. PyMOL (ver 2.3.0) (https://pymol.org) rendered the cartoon diagrams, and the APBS plugin within the software was used to calculate the electrostatic surface potential. A cubic box with sides roughly 10 Å away from the protein was used with SPC/E (SPC216) water as the solvent. The aqueous protein system was neutralized and minimized to remove steric clashes using the steepest descent method such that the maximum force in the system was not more than 1000 kJ/mol/nm. Subsequently, we performed NVT equilibration, with a 2 fs time step, deploying the modified Berendsen thermostat with a total simulation time of 100 ps under a temperature of 300 K. Next, we performed the NPT (constant number (N), pressure (P) and temperature (T) equilibration of 100 ps using a 2 fs time step at 1 atm using the Parinello-Rahman pressure coupling. The *in silico* docking of ATP was performed onto two suitable frames for Hop1 structure using Autodock Vina (80). To achieve this, a 3D map of the grid box was defined (in the conf.txt file) that enclosed the complete Hop1 molecule, such that the ATP docking scan encompassed the whole protein surface (blind docking). The ATP and Hop1 molecule coordinates were saved in the pdbqt format and copied into a folder, following which the Vina program was run using the command prompt, at an exhaustiveness value of 300.

### Site-directed mutagenesis

The mutant alleles that express different variants of Hop1 protein were constructed by performing site-specific mutagenesis using the construct pET28a-*HOP1* as a template (81). In particular, substitution of aa residues in the putative ATP-binding pocket of Hop1 (K65A, N67Q, R352A, R558A and N139Q) were generated by overlapping Polymerase chain reaction (PCR) method (82). The forward and reverse primers that were used to amplify the *HOP1* coding sequence harboring the respective substitutions are mentioned in Supplementary Table S1. The PCR reactions contained 100 ng of plasmid DNA, 1X Phusion buffer, 2% Dimethyl sulfoxide (DMSO), forward and reverse primer (2 μM each), 100 μM dNTPs and 50 μM MgCl_2_. Each PCR cycle was characterized by initial denaturation at 95°C for 5 min, annealing at 60°C (40 s), initial extension at 72°C (2 min), and final extension at 72°C (10 min). Following PCR, 5 μg of the amplified DNA and pET28a vector were digested with NdeI/XhoI at 37°C for 6 h. The desired DNA fragment was extracted from the gel and ligated with the vector using T4 DNA ligase at 16°C for 10 h. The ligation mixture was transformed into *E. coli* TOP10 cells (Invitrogen) and selected on Luria-Bertani (LB) plates supplemented with kanamycin (50 μg/ml). Analogously, the double mutants, *hop1-K65A,N67Q* and *hop1-R352A,R558A*, were constructed using the corresponding single mutant plasmids (pET28a-*hop1-K65A* and pET28a-*hop1-R352A*) as templates. The identity of all the mutants was confirmed by restriction digestion and DNA sequencing. The plasmid pET21a-Hop1-CTD has been described previously (61). The nucleic acid sequence encoding Hop1-HORMA domain was cloned and expressed using the pET14b vector. For this purpose, the sequence corresponding to Hop1-HORMA was amplified using the pET28a-*HOP1* plasmid as the template. The resulting DNA fragment was digested with NdeI/XhoI and ligated into the NdeI/XhoI digested pET14b vector.

### Expression and purification of WT and variant Hop1 proteins

The genes encoding WT and single/double amino acid substitution variants of Hop1 (Hop1^K65A^, Hop1^N67Q^, Hop1^K65A,N67Q^, Hop1^N139Q^, Hop1^R352A^, Hop1^R558A^ and Hop1^R352A,R558A^) were cloned into the pET-28a vector and expressed, as described previously with few modifications (81). The Ni^2+^-NTA resin and size-exclusion chromatography were used for the purification of WT Hop1. Briefly, the *E. coli* BL21*(DE3) cells harboring the pET28a-*HOP1* plasmid were grown overnight in LB broth supplemented with kanamycin (50 μg/ml) at 37°C, with shaking at 180 rpm. When the cultures reached OD_600_ of 0.5, IPTG was added to a final concentration of 0.5 mM to induce protein expression, and the cultures were incubated for 10 h at 18°C, with shaking at 180 rpm. The cells were harvested and resuspended in Buffer A (50 mM HEPES, pH 7.5; 250 mM NaCl, 5% glycerol, 1 mM each of phenylmethylsulfonyl fluoride [PMSF] and benzamidine). The cells were lysed by sonication (Amplitude, 55%; pulse on, 1s; pulse off, 1 s; time, 2 min; 6 cycles). The cell debris was removed by centrifugation using a Beckman Ti-45 rotor, at 70 400 × *g* for 1.5 h. The supernatant was loaded onto a 10 ml Ni^2+^-NTA column, pre-equilibrated with Buffer A. The column was washed with Buffer A containing 60 mM imidazole and 500 mM NaCl. This step effectively removed all the impurities, including DNA and NTPs. The bound protein was eluted using an imidazole gradient of 70–800 mM in buffer A and the individual fractions were analyzed using 10% SDS-PAGE and protein bands were visualized by Coomassie blue staining. The fractions containing Hop1 protein were pooled, concentrated to 5 ml and loaded onto a gel filtration column (Superdex 200 10/30 GL, GE Healthcare), connected to the Bio-Rad Biologic Duoflow chromatography system. The bound protein was eluted in Buffer A containing 500 mM NaCl, and the fractions corresponding to the peak were analyzed on 10% SDS-PAGE, and protein bands were visualized as described above. The Hop1-CTD and Hop1 single and dual amino acid substitution variants (Hop1^K65A^, Hop1^N67Q^, Hop1^K65A,N67Q^, Hop1N^139Q^, Hop1^R352A^, Hop1^R558A^ and Hop1^R352A,R558A^) were purified to homogeneity using the same protocol as described for the WT Hop1. The final preparations were dialyzed against buffer A and stored at –80°C.

The nucleic acid sequences encoding Hop1-CTD and Hop1-HORMA domains were cloned, transformed into a Rosetta (DE3) *E. coli* strain and expressed using the pET-21a and pET-14b vector, respectively. The Hop1-CTD was expressed and purified as previously described (61). To purify Hop1 HORMA domain, cells were grown in LB broth supplemented with ampicillin (50 μg/ml) at 37°C, with shaking at 180 rpm. When the culture reached OD_600_ of 0.5, protein expression was induced by the addition of IPTG to a final concentration of 0.5 mM, and incubation was extended for 12 h at 18°C, with shaking at 180 rpm. The cells were harvested and resuspended in Buffer B (25 mM Tris–HCl pH 8.0, 400 mM NaCl, 20% glycerol, and 0.2% sarkosyl) and lysed by sonication, as described above. The cell debris was removed by centrifugation at 70 400 × *g* for 1.5 h, and the pellet was discarded. After the addition of Triton X-100 to the supernatant to a final concentration of 0.5%, it was applied onto a Ni^2+^-NTA agarose column that had been pre-equilibrated with buffer B minus sarkosyl. The column was washed with 10 bed volumes of Buffer B (containing 50 mM imidazole), and the bound protein was eluted using an imidazole gradient (60→800 mM) in Buffer B. Fractions containing Hop1 HORMA domain was analyzed on 10% SDS-PAGE, pooled, dialyzed against Buffer A and stored at –80°C. The purified proteins were found to be >95% pure, as judged by SDS-PAGE and Coomassie blue staining, and their concentrations were determined using BSA as a standard (83).

### Cell-free transcription, expression and purification of WT Hop1

The *HOP1* mRNA was produced using the Ribomax T7 (Promega) *in vitro* transcription system. The plasmid bearing the WT *HOP1* gene under the control of the T7 promoter was linearized and used for the transcription reaction. The total RNA was isolated using the TRIzol method, based on the manufacturer’s instructions (Invitrogen, Santa Clara, CA). The reaction mixture containing rabbit reticulocyte lysate (Promega), 20 μM complete amino acid mix, 2 μg isolated RNA and 0.8 U/μl RNA polymerase was incubated at 37°C for 3 h. To the same reaction mixture, we added 200 μl of buffer (20 mM Tris–HCl, pH 8, 10% glycerol, 400 mM NaCl, and 5 mM 2-mercaptoethanol) and pre-equilibrated Ni^2+^-NTA agarose slurry, and incubated at 4°C for 16 h with gentle shaking. Subsequently, the Ni^2+^-NTA beads were collected by centrifugation, washed with 500 μl of buffer containing 20 mM Tris–HCl (pH 7.5), 10% glycerol, 400 mM NaCl, 20 mM imidazole and 5 mM 2-mercaptoethanol. The bound protein was eluted stepwise with 150, 250 and 500 mM imidazole in the elution buffer (20 mM Tris–HCl, pH 7.5, 10% glycerol, 1 mM EDTA, 40 mM NaCl and 5 mM 2-mercaptoethanol). An aliquot from every other fraction was analyzed by SDS-PAGE, and the proteins were visualized by Coomassie blue staining. The fractions containing Hop1 were pooled and stored at –80°C.

### Immunoblotting

Western blot analysis was performed as previously described (84). The WT Hop1 and its variants (Figure 5C) were subjected to 10% SDS-PAGE, and the proteins were transferred from the gel onto immobilion polyvinylidene fluoride (PVDF) membrane (Merck Life Sciences, Bangalore) at 130 V for 1 h. Following the transfer, the membrane was blocked with 5% skimmed milk in TBST buffer (20 mM Tris–HCl, pH 8; 150 mM NaCl, 0.5% Tween-20) at 25°C for 1 h. The membrane was washed with TBST buffer and incubated in anti-Hop1 antibody solution diluted to 1:10 000 with gentle shaking. After 12 h incubation at 4°C, the membrane was washed three times with TBST buffer and incubated with anti-rabbit antibodies diluted to 1:40 000 (in TBST) for 4 h at 4°C, followed by washing twice with TBST buffer. The antibody-stained protein band was visualized with horseradish peroxidase (HRP)-conjugated secondary antibody (Clarity Western ECL substrate, Bio-Rad #170–5061)). The image was captured using the ImageQuant LAS 4000 imager (Cytiva).

Immunoblotting of the *S. cerevisiae* whole cell lysates was performed as described previously (37) with minor modifications. Approximately 8 × 10^7^ cell samples were collected at different time points after transfer into the sporulation medium and subjected to centrifugation at 3220 × *g* for 5 min. The cell pellets were resuspended in 200 μl of 20% trichloroacetic acid (TCA) and 200 μl of acid-washed glass beads. The cells were lysed by vortexing for 3 min (×5) bursts at 4.0 m/s using a FastPrep-24 homogenizer (MP Biomedicals). To each sample, 200 μl of 5% TCA was added, and samples were vortexed for 2 min. The precipitated proteins were collected by centrifugation at 3381 × *g* for 3 min. The pellets were resuspended in 40 μl of Tris–HCl buffer (pH 9.1) and 100 μl 2X buffer [4% SDS, 120 mM Tris–HCl (pH 6.8), 1% bromophenol blue and 5 mM DTT]. The samples were denatured by incubating at 95°C for 10 min, followed by centrifugation at 9391 × *g* for 30 s. The aliquots (25 μl) of each sample were resolved on a 10% SDS-PAGE gel, and the proteins were transferred to a PVDF membrane as previously described (84). The membranes were probed with either 1:10 000 dilution of rabbit-raised anti-Hop1 antibodies or 1:5000 dilution of mouse anti-Pgk1 antibodies (Abcam Ltd., AB113687) for 12 h at 4°C. The membranes were washed thrice in TBST buffer and incubated with 1:15 000 dilution of either anti-rabbit or anti-mouse antibodies, for 4 h at 4°C. The blots were developed as described above.

### Thermal stability assay

The assay was performed with SYPRO Orange using a 96-well plate in a Bio-Rad CFX real-time PCR detection system, which allows simultaneous analysis of protein variants as previously described (85). The reaction mixtures (20 μl) consisting of 25 mM HEPES, pH 7.5, 2 μl 5X SYPRO Orange (Sigma-Aldrich), 10 μg of WT Hop1 or its variants, with or without 5 mM NTPs were transferred into a 96-well PCR plate. It was sealed with microseal PCR plate sealing film (Bio-Rad) and centrifuged briefly to remove air bubbles. The thermal stability was monitored by heating the samples from 25 to 95°C at a rate of 0.5°C/min. The fluorescence emission spectra were recorded at 570 nm at 30 s intervals, and *T_m_* was calculated using the GraphPad Prism software. The relative fluorescence (RFU) and the derivative values [-d(RFU)/dT], as a function of temperature (°C), were obtained using the CFX Maestro software (version 2.3). The thermal unfolding curves were generated using Graph Pad Prism (version 5.0).

### Isothermal titration colorimetry

The measurements were carried out at 20°C using a VP-ITC calorimeter (MicroCal Northampton, MA) as described previously (86–88) with some modifications. Prior to the isothermal titration colorimetry (ITC) experiments, WT Hop1 and the Hop1^K65A,N67Q^ variant were dialyzed overnight against a buffer comprising of 50 mM HEPES (pH 7.5), 250 mM NaCl and 5% glycerol. The ATP was dissolved in the same buffer. The syringe was filled with 500 μl of 0.6 mM ATP and the reference cell was filled with 2 ml of protein solution. Each experiment was set to 30 injections (10 μl each) of ATP into the protein containing sample cell with 240 s intervals and a filter period of 2 s. The stirring speed was set at 307 rpm at a reference power of 10 μcal/s. The data were integrated to generate binding curves, following which the areas under the injection peaks were plotted against the molar ratio of ATP to protein. Analysis of the data was performed using the MicroCal Origin software. The curve was fitted into the one site binding model to obtain the dissociation constant and thermodynamic parameters. The heats of dilution were determined from control experiments and were subtracted from the binding data prior to curve fitting.

### ATP-agarose affinity chromatography

Purified Hop1 WT and the Hop1^K65A,N67Q^ variant (1 mg/ml) were individually loaded at 4°C onto 2 ml ATP-agarose columns (Sigma-Aldrich), which had been equilibrated with buffer A (50 mM HEPES [pH 7.5] 250 mM NaCl, 5% glycerol and 1 mM DTT). The flow-through and column wash were collected in fractions. The bound proteins were eluted from the column by applying an ATP gradient from 5 to 50 mM in buffer A. The eluted fractions were analyzed by 10% SDS–PAGE and the proteins were visualized by staining with Coomassie brilliant blue.

### Preparation of DNA substrates

The DNA substrates used in this study are shown in Supplementary Table S2. The corresponding ODNs (Supplementary Table S3) were labeled at their 5′-ends with [γ-^32^P]ATP and T4 polynucleotide kinase as previously described (64,89). The labeled ODNs were separated from free [γ-^32^P]ATP by passing the reaction mixture through a Sephadex G-50 column (G-Biosciences). The labeled ODNs retained on the column were eluted with TE buffer (10 mM Tris, pH 7.5, 1 mM EDTA). The HJ and dsDNA substrates (Supplementary Table S2) were constructed by annealing the appropriate combinations of unlabeled and ^32^P-labeled ODNs in a solution containing 0.3 M sodium citrate buffer (pH 7.0), followed by incubation at 95°C for 3 min and cooling at 20°C for 4 h. The annealed substrates were separated on an 8% polyacrylamide gel under non-denaturing conditions. The corresponding DNA bands were excised from the polyacrylamide gel, and the DNA was eluted from the gel pieces using 1× TE buffer (10 mM Tris–HCl containing 1 mM EDTA, pH 8.0). To construct the 5′-FAM labeled HJ, equimolar concentrations of 5′-FAM HJ02, HJ01, HJ03 and HJ04 were mixed, incubated and isolated as described above.

### ATPase assays

The hydrolysis of [γ-^32^P]ATP by WT Hop1 and its variants was measured by a radiometric assay using thin-layer chromatography. The assay was carried out in a buffer (20 μl) consisting of 50 mM Tris–HCl (pH 7.5), 200 pM [γ-^32^P]ATP, 50 or 20 μM ATP (also contains 200 pM [γ-^32^P]ATP as a tracer), and various concentrations of WT Hop1 or its variants, in the absence or presence of 500 nM of ssDNA, dsDNA or the HJ. After incubation at 37°C for 30 min, the reactions were stopped by adding 2 μl of a stop buffer (1 mg/ml proteinase K, 2.4% w/v SDS and 100 mM EDTA). An aliquot (2 μl) from each reaction was removed and spotted on a polyethyleneimine (PEI) pre-coated sheet (Machery-Nagel, Düren, Germany), and developed using a solution containing 1 M formic acid and 0.5 M LiCl. The radiolabeled spots were visualized using a Fuji FLA-9000 phosphorimager and quantified in UVItec gel documentation system using the UVIBand map software (version 97.04). Graphs and statistical analyses were generated using Graph Pad Prism (version 5.0).

A malachite green-based colorimetric assay was used to measure the release of inorganic phosphate (P_i_), as previously described (90). The assay was performed in an 80 μl reaction mixture containing 50 mM HEPES (pH 7.5) and 500 nM DNA cofactor (where specified), a fixed amount of ATP and different concentrations of WT Hop1 or its variants. After incubation for the indicated times at 37°C, the reactions were stopped by adding 20 μl of malachite green solution (98% v/v Reagent A + 2% (v/v) Reagent B) (Sigma-Aldrich), followed by an additional 20 min incubation at 25°C. The amount of P_i_ released was determined by measuring the absorbance at 620 nm using the VersaMAX microplate reader (Molecular Devices, Sunnyvale, CA, USA). The time course experiments were performed by incubating a fixed concentration of Hop1 (200 nM) with a fixed concentration of ATP (100 μM) and assayed as described above. The phosphate released in the reactions was quantified with an internal calibration curve constructed using inorganic phosphate as specified in the kit. The reaction velocity was calculated at each ATP concentration using the formula, velocity = [phosphate]/time of reaction. The *K*_m_ and *V*_max_ values were obtained by linear regression of the Lineweaver-Burk plot using GraphPad Prism (version 5.0).

### Electrophoretic mobility shift assays

The EMSAs were performed as previously described (61,64) with a few modifications. The reactions were carried out in a 20 μl reaction mixture consisting of 25 mM HEPES (pH 7.5), and 0.5 nM of ^32^P-labeled HJ with varying concentrations of WT and its variants. After incubation at 37°C for 15 min, the reactions were stopped by adding a 2 μl of stop solution (0.25% bromophenol blue, 0.25% xylene cyanol, 20% glycerol). The reaction mixtures were separated at 4°C by non-denaturing electrophoresis through an 8% polyacrylamide gel at 80 V using the TBE buffer (25 mM Tris base, pH 8.0, 25 mM boric acid and 0.5 mM EDTA) for 5 h. The gels were dried and exposed to the phosphor imager screens for 12–14 h. The bands were visualized in a Fuji FLA-9000 phosphorimager (GE Healthcare), and the band intensities were quantified using the UVIBand map software (version 97.04). The data were fitted into nonlinear regression analysis using the equation *Y* = (*B*_max_ * *X*)/(*K*_d_ + *X*) to obtain the apparent dissociation constant (*K*_d_) values, from three independent experiments. The graphs were generated using GraphPad Prism (version 5.0).

### Microscale thermophoresis

The MST measurements were carried out using a Monolith NT.115 instrument (NanoTemper) at 25°C. The experiments were performed in a buffer consisting of 25 mM HEPES (pH 7.5), 25 nM of 5′-FAM labeled HJ and varying concentrations of WT or the Hop1^K65A,N67Q^ variant (0.061 nM→2 μM), as previously described (91,92). After incubation at 37°C for 15 min, the samples were loaded into Monolith NT.115 glass capillaries, and the MST analysis was performed with blue light (with 40% excitation power and 40% MST power). The fluorescence change in MST signal is normalized (*F*norm[%]) was plotted against the concentration of WT and Hop1^K65A,N67Q^ variant to obtain the dose–response curve. The NanoTemper analysis software was used to fit the data to determine the apparent *K*_d_ values. The graph was generated using the GraphPad Prism software (version 5.0).

The interaction between Hop1-CTD and HORMA domain was ascertained by mixing fixed concentrations of Red-tris-NTA labeled Hop1 HORMA domain (20 nM) with various concentrations of Hop1-CTD (0.00031 to 10 μM). After incubation at 37°C for 15 min, samples were transferred to standard MST Monolith glass capillaries. The measurements were recorded at 5% excitation power using Pico-RED excitation type. The fraction bound (*X*) obtained using the equation *X*= [*Y*(*c*) − Min]/(Max – Min), was plotted against increasing Hop1-CTD concentration to obtain the binding isotherms. Equilibrium dissociation constants (*K*_d_) were calculated using Hill model in the Nanotemper Monolith Affinity Analysis software and graphs were obtained using GraphPad Prism software (v5.0).

### Nitrocellulose filter binding assay

The assay was performed by incubating various concentrations of Hop1 with 0.5 nM of [α-^32^P]ATP in buffer A (50 mM HEPES, pH 7.5, 100 mM NaCl, 20% glycerol, 5 mM 2-mercaptoethanol) for 10 min at 30°C. Subsequently, the samples were exposed to a UV source (1.2 × 105 μJ/cm^2^) at a distance of 2 cm, for 10 min using the Hoefer UVC500 Ultraviolet cross-linker. Similarly, the reactions were performed with fixed concentrations of the *S. cerevisiae* Rev7 (*Sc*Rev7) and *Mycobacteruim tuberculosis* RecA (*Mt*RecA) proteins. Aliquots (5 μl) of each sample were spotted on a nitrocellulose membrane (0.45 mm pore size, Bio-Rad Laboratories) and then air-dried at 25°C. The membrane was washed using ice-cold buffer A (containing 200 mM NaCl) for 5 cycles (5 min each) to remove the unbound [α-^32^P]ATP. The dried membrane was exposed to a phosphorimager screen for 14 h. The radioactive spots were visualized using a Fuji FLA 9000 phosphorimager, and the spot intensities were quantified using the ImageJ software (Ver 1.53). The graphs were generated by performing non-linear regression analysis of the data using the GraphPad Prism software (Ver 5.0).

### Immunodepletion assay

The assay was performed by incubating 3 μg of Hop1 with 1 μl of anti-Hop1 antibodies (2.5 mg/ml), followed by 50 μl of protein A-Sepharose beads (Cytiva) at 4°C for 4 h in a binding buffer (20 mM Tris–HCl, pH 7.5, 25% glycerol, 300 mM NaCl and 5 mM 2-mercaptoethanol). The pellet and supernatant fractions were separated by centrifugation at 3220 × *g* for 5 min at 4°C. A sample from the pellet fraction, solubilized in 1× Laemmli buffer (65 mM Tris–HCl [pH 6.8], 0.1% 2-mercaptoethanol, 0.0005% bromophenol blue, 10% glycerol and 2% SDS), was subjected to 10% SDS-PAGE, and the protein bands were visualized by Coomassie blue staining. The pellet and supernatant fractions were analyzed for ATPase activity using malachite green to assay as described above. The graph was generated using the GraphPad Prism (ver 5.0). The error bars represent the deviation across three independent experiments.

### Far western blot analysis

The assay was performed by spotting increasing concentrations of *Ms*RecA or untagged Hop1-CTD onto nitrocellulose membranes and air dried. Membranes were blocked with a buffer containing 25 mM HEPES, pH 7.5, 100 mM NaCl, 1 mM EDTA and 5% skimmed milk (w/v) for 2 h at 25°C. The membranes were incubated with purified Hop1 HORMA diluted in blocking buffer for 12 h at 4°C and washed thrice with a buffer B (25 mM HEPES, pH 7.5, 100 mM NaCl and 1 mM EDTA) containing 0.005% Nonidet P-40. The membranes were then incubated with anti-His antibodies (1:7000) for 7 h at 4°C, washed with buffer B and finally incubated with HRP-conjugated anti-rabbit antibodies for 4 h at 4°C. Chemiluminescence signals were detected using Bio-Rad ChemiDoc imaging system.

### Yeast strains and plasmids

The sequence of primers used to generate the yeast strains in this study are listed in Supplementary Table S4. *S. cerevisiae* strains (listed in Supplementary Table S5) were cultured in either yeast peptone dextrose (YPD) or YPL [1% (w/v) yeast extract, 2% (w/v) peptone and 2.5 ml lactic acid (85% stock solution)/100 ml (pH 5.5)] or synthetic medium as previously described (93). The haploid KTY81 and KTY82 cells were mixed and grown overnight in 3 ml of YPD medium (composed of yeast extract, peptone and dextrose) at 30°C, and plated on YNB (-Trp, -Ura) medium for selection of the resulting diploid (KMY54). To delete *HOP1* gene, a linear DNA cassette containing sequences flanking the gene and the sequence corresponding to the hygromycin resistance cassette (from the plasmid pFA6a-hphNT1) were generated using the primers FPDelHop1 and RPDelHop1 (Supplementary Table S4), as previously described (94). The resulting DNA fragment was PCR-amplified using two-step cycles of denaturation and annealing plus elongation (30 cycles of denaturation at 95°C for 5 min, annealing at 55°C for 40 s plus extension at 72°C for 2 min). The PCR product was transformed into KTY81 and KTY82 cells and selected on solid YPD medium containing 300 μg/ml hygromycin B, as previously described (95). The genomic DNA of the transformants was PCR-amplified using the primers Locus FP and RPDelHop1 to verify integration at the site-specific locus. The gene deletion was confirmed by DNA sequencing in both KMY63 and KMY56. The strain KMY 64 (SK1 *hop1Δ/hop1Δ*) was generated by mating the KMY63 and KMY56 haploids. The same strategy was used to delete the *HOP1* gene in the NHY1162 and NHY1168 background to construct KMY69 and KMY70 strains (Supplementary Table S5).

To construct a strain with *hop1-K65A,N67Q* allele, the G418-resistance marker gene was amplified using the pFA6a-kanMX4 plasmid (primer pair FPKanMX4 and RPDelHop1). Analogously, the *hop1-K65A,N67Q* allele was amplified using pET28a-*hop1-K65A,N67Q* as a template using the primer pair FPHop1 cassette and RP*HOP1*-KanMX4, as previously described (94,96). A 3.4-kb cassette containing the *HOP1* gene with K65A,N67Q substitutions and G418 resistance marker was constructed by overlapping PCR with these DNA fragments using the primers FPHop1cassette and RPDel*HOP1* (Supplementary Table S4). The resulting DNA fragment was transformed into haploid strains to generate KMY65, KMY66, KMY72 and KMY73 strains. The genomic DNA obtained from the transformants, grown on YPD medium containing 200 μg/ml G418, was PCR-amplified using primers Locus FP and RPDel*HOP1* to verify integration at the site-specific locus. Diploid SK1 (KMY68 and KMY74) strains were constructed by mating the MATa and MATα haploids (Supplementary Table S5). The strains were confirmed by Sanger sequencing. The genetic distances were analyzed using *S. cerevisiae* strains KMY54 and KMY68 or the derivatives. The ChIP-seq analyses were performed using NHY1162/1168 and the derivative strains as previously described (45).

### Meiotic progression analysis by DAPI staining

The meiotic time-course experiments were conducted with the strains KMY54, KMY64 and KMY68. Briefly, single colonies from freshly streaked YPL agar plates were grown in YPD liquid medium at 30°C for 16 h, with gentle shaking. Aliquots of these cultures were inoculated into a 25 ml pre-sporulation medium consisting of 0.5% w/v yeast extract, 1% w/v bactopeptone, 0.67% w/v yeast nitrogen base, 1% potassium acetate, and 1% potassium bipthalate (pH 5.5) and grown until the OD_600_ reached 0.8. Then, the cells were inoculated into a 225 ml pre-sporulation medium at a starting OD_600_ of 0.05 and grown at 30°C for 16 h with gentle stirring at 300 rpm. The cells were collected by centrifugation at 3220 × *g* for 5 min and washed twice with a pre-warmed (30°C) sporulation medium (2% potassium acetate). The pellet was resuspended in a 150 ml sporulation medium by gentle shaking. Aliquots of the cells were removed at the indicated time intervals and centrifuged at 3381 × *g* for 5 min. The cell pellets were resuspended in 40% aqueous ethanol and stained with DAPI. Image analysis was performed using a The Olympus Fluoview FV3000 laser scanning confocal microscope and FV31S-SW version 2.4.1.198 software. At least 100 cells were counted for each time point to assess the percentage of cells that have completed meiosis I and meiosis II. The curves were generated using GraphPad prism (version 5.0).

### Spore viability and tetrad analysis

The haploid WT and *hop1* mutants in the KTY81/KTY82 strain background were grown in YPD medium as previously described (97,98). The haploids were mated on a synthetic complete medium (98) for 4 h at 30°C, and the resulting diploid strains were sporulated on a sporulation medium as previously described (99,100). A tetrad dissection was performed on the SC medium using a Zeiss dissection microscope. The dissected spores were incubated for 2–3 days at 30°C to allow germination and develop into visible colonies. The viability of spores was calculated based on the percentage of spores surviving per tetrad class for each strain. To determine the genetic map distance, the spore clones were replica-plated onto drop-out plates, and the growth was assessed after 14 h of incubation. The genetic map distances between the markers were estimated from the segregation data using the RANA software, from both the tetrads as well as the single spore analysis as previously described (100). Statistical analysis was performed using the Stahl Laboratory Online Tools (https://elizabethhousworth.com) and VassarStats (http://vassarstats.net/). The data shown represent values from experiments performed using two independent transformants. The *P*-values were obtained by performing the two-tailed G-tests (https://www.biostathandbook.com/gtestgof.html).

### Chromatin immunoprecipitation

The *HOP1/HOP1*, *hop1-K65A,N67Q/hop1-K65A,N67Q* and *hop1Δ/hop1Δ* strains in the NHY1162/NHY1168 strain background were inoculated into 4 ml of YPD medium and grown at 30°C for 14 h. Meiotic synchronization was carried out as previously described (101). Briefly, ∼5 × 10^6^ cells/ml (OD_600_ = 0.8) from overnight YPD cultures were inoculated into 25 ml of SPS medium (0.5% yeast extract, 1% peptone and 0.67% yeast nitrogen base without amino acids, 0.05 M potassium biphthalate, pH 5.5 and 1% potassium acetate) containing 1% antifoam (1 μl/ml). The cultures were grown at 30°C for 6 h. From these cultures, ∼3 × 10^5^ cells/ml (OD_600_ = 0.05) were inoculated into 300 ml of SPS medium and grown at 30°C for 16 h to a density of ∼3–4 × 10^7^ cells/ml (OD_600_ = 4.5). The cells were harvested by centrifugation at 3220 × *g* for 4 min, washed twice with 2% potassium acetate and resuspended to a cell density of 4 × 10^7^ cells/ml in 200 ml of SPO (2% potassium acetate) medium supplemented with amino acids and polypropyl glycol (1 μl/ml). The kinetics of meiotic cell cycle progression was monitored by DAPI nuclear staining, followed by visualization under a fluorescence microscope.

We performed calibrated ChIP-seq profiling of WT Hop1 and Hop1^K65A,N67Q^ variant using the ChIP-grade Hop1 antibody and protein A-Sepharose beads (GE Healthcare), as previously described (45,101–104) with minor modifications. Fifty milliliters of *S. cerevisiae* meiotic cultures, after 4 h incubation, were mixed with five ml of *Saccharomyces mikatae* culture at the same time after meiosis induction. Both the cultures were mixed, treated with 1% formaldehyde for 30 min, and then glycine was added to a final concentration of 125 mM to quench formaldehyde. The cells were harvested by centrifugation (8000 rpm, 4°C, 5 min) and washed twice with 40 ml of TBS (50 mM Tris–HCl with 150 mM NaCl, pH 7.6). The resulting cell pellets were kept at −80°C. The cells were lysed using a bead beater (Biospec, Bartlesville, OK, USA). The lysates were further sonicated using a Bioruptor Twin (Diagenode). Cell lysates were pre-cleared by incubation with a suspension of protein A-Sepharose beads. The pre-cleared extract was incubated with anti-Hop1 antibody with gentle agitation at 4°C overnight for 14 h, and then with protein A-Sepharose beads. The supernatants were removed by centrifugation, followed by washing the beads with TBS to remove nonspecific binding proteins. The immunoprecipitated DNA was collected by centrifugation at 4°C. The resuspended sample was incubated with 1 μl/ml RNase A (Sigma-Aldrich) at 65°C for 14 h. DNA was treated with proteinase K (20 mg/ml, HIMEDIA). The DNA fragments was purified using a Qiagen PCR purification kit (#28 106) and sequenced using the Illumina Novaseq 6000 platform (San Diego, USA) by an established service provider (Macrogen Inc., Seoul, Korea).

### Calibrated ChIP-seq data analysis

The calibrated ChIP-seq data were analysed as previously described (45,102–104). FastQC was used to check the quality of the raw sequencing data obtained for the *HOP1/HOP1*, *hop1-K65A,N67Q/hop1-K65A,N67Q* and *hop1Δ/hop1Δ* strains (https://www.bioinformatics.babraham.ac.uk/projects/fastqc/). The reads from these samples were aligned separately to the *S. cerevisiae* S288c (version 64–1-1, 2011) and *S. mikatae* IFO1815 (GCF_947241705.1) genomes using Bowtie2 (version 2.3.5.1; (105)). Uniquely mapped reads for *S. cerevisiae* were obtained by aligning the unmapped *S. mikatae* reads to the S288c reference genome. Similarly, uniquely mapped reads for *S. mikatae* were obtained by aligning the unmapped *S. cerevisiae* reads to the *S. mikatae* IFO1815 spike-in genome. The occupancy ratio for Hop1 binding was calculated using these uniquely mapped reads as previously described (103). The S288c genome was partitioned into 10 bp bins to calculate RPM (reads per million) normalized Hop1 reads. These were multiplied by the occupancy ratio to obtain the calibrated Hop1 reads at each genomic bin. The calibrated Hop1 read depths were averaged from two biological replicates per strain and were subsequently smoothened using the R function ksmooth. The binding plots for *HOP1/HOP1* and *hop1-K65A,N67Q*/*hop1-K65A,N67Q* strains were generated in R and correlation was determined using the Karl Pearson’s correlation coefficient. The statistical significance for the boxplots was calculated using the Wilcoxon rank sum test.

### ChIP-qPCR analysis

The assays were performed in 96-well semi-skirted PCR plates (Bio-Rad, #HSS9601) using the TB Green Premix Taq II (Tli RNaseH Plus, # RR820A) and the QuantStudio 1 real-time PCR system (Thermo Fisher Scientific), as previously described (102). Approximately 100 ng DNA from the ChIP and input samples of *HOP1/HOP1, hop1-K65A,N67Q/hop1-K65A,N67Q* and *hop1Δ/hop1Δ* strains was used for qPCR (quantitative polymerase chain reaction) analyses. The primer sequences (Sigma-Aldrich) for qPCR are listed in Supplementary Table S10. The binding of Hop1 WT and Hop1^K65A,N67Q^ variant was analyzed at DSB hotspots (*BUD23*, *ECM3*), axes (Axis I, Axis III), centromere (*CEN VIII*) and DSB cold spot (*YCR093W*) using the primer sets listed in Supplementary Table S10. The qPCR reaction conditions consisted of an initial denaturation at 95°C for 3 min, followed by denaturation at 95°C for 10 s, annealing at 55°C for 5 s and a melting curve set at 65°C for 5 s. Finally, qPCR was performed at 95°C for 5 s to confirm amplification of a single PCR product. The fold enrichment of the ChIP DNA was calculated relative to the amount of DNA in the corresponding input sample, and normalized to the DSB cold spot. All measurements were compared to a standard curve generated from a dilution series of the corresponding input samples. The Ct values were extracted using QuantStudio design and analysis software (v1.5.1).

### Web resources

The following web-based software resources have been used in this study. C-I-Tasser (https://zhanggroup.org/C-I-TASSER/), Stahl Laboratory Online Tools (https://elizabethhousworth.com/) VassarStats webpage (http://vassarstats.net/), Cofactor server (https://zhanggroup.org/COFACTOR/) and G-tests (http://www.biostathandbook.com/gtestgof.html).

## Results

### Discovery of Hop1 ATPase activity

**Figure 1.**
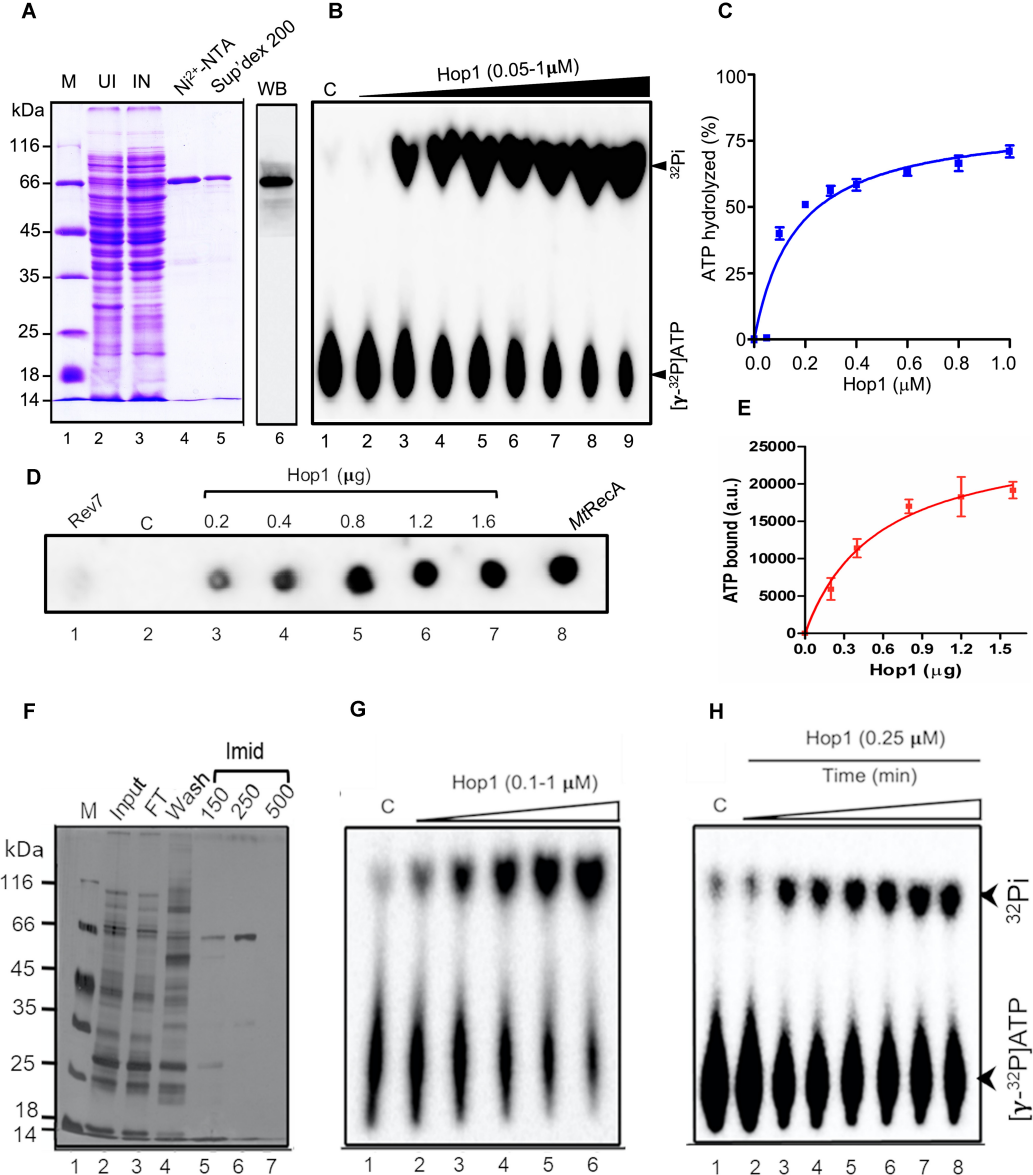
Hop1 has intrinsic ATPase activity. (**A**) A Coomassie blue-stained SDS-PAGE gel showing analysis of protein samples at different stages of purification of Hop1. Lane 1, molecular weight standards; lane 2, whole-cell lysate from uninduced cells (25 μg); lane 3, whole-cell lysate from induced cell lysates (25 μg); lane 4, eluate from Ni^2+^-NTA affinity column (5 μg); lane 5, eluate from Superdex 200 column (3 μg); lane 6, western blot analysis of purified Hop1 (fraction 5) using anti-Hop1 antibodies. (**B**) A thin-layer chromatogram showing [γ-^32^P]ATP hydrolysis by Hop1 in a concentration-dependent manner. Increasing concentrations of Hop1 were incubated with 20 μM cold ATP (and 200 pM [γ-^32^P]ATP as a tracer) at 37°C for 40 min. The reaction products were analyzed by TLC. (**C**) Graph shows quantification of the ATPase activity (*n* = 3). (**D**) Hop1 binds to [α-^32^P]ATP. The reaction mixtures containing indicated proteins were incubated with 0.5 nM [α-^32^P]ATP. Samples were analyzed using a nitrocellulose filter binding assay. Lane 1, *S. cerevisiae* Rev7 (1 μg); lane 2, No protein (control). lanes 3–7, various concentrations of Hop1 (0.2, 0.4, 0.8, 1.2 and 1.6 μg), and lane 8, MtRecA (0.4 μg). (**E**) The graph shows the quantification of [α-^32^P]ATP-cross-linked species (*n* = 3). (**F**) Purification of Hop1 expressed in the cell-free translation system. Samples at various stages of purification of Hop1 were analyzed by SDS-PAGE and staining with Coomassie blue. Lane 1, molecular weight standards; lane 2, 5 μg protein of the translation mixture (input); lane 3, Ni^2+^-NTA column flow-through (5 μg); lane 4, column wash with 20 mM imidazole (5 μg). Lanes 5–6, protein eluted with 150 and 250 mM imidazole. Lane 7, elution with 500 mM imidazole (Imid). (**G**) A thin-layer chromatogram showing Hop1 made in the cell-free protein synthesis system catalyses [γ-^32^P]ATP hydrolysis. Various concentrations of Hop1 (0.1–1 μM) were incubated with 20 μΜ cold ATP (and 200 pM [γ-^32^P]ATP as a tracer) for 30 min at 37°C. (**H**) A thin-layer chromatogram showing time course of ATP hydrolysis by Hop1 from the cell-free protein synthesis system. Lane1, reaction performed in the absence of Hop1. Lanes 2–8 correspond to increasing reaction times as follows: 5, 10, 15, 20, 30, 45 and 60 min, respectively. The reaction products were analyzed as in panel (B). (B, G and H) 2 μl from each reaction mixture was spotted on a TLC plate and developed in a solution containing 1 M HCOOH, 0.5 M LiCl and 1 mM EDTA. The TLC plates were dried and imaged using a Fuji FLA-9000 phosphorimager.

We attempted to determine a three-dimensional structure of *S. cerevisiae* Hop1 via X-ray crystallography but these attempts have not been successful. Thus, we employed a triad of experimental approaches, namely, *in silico*, *in vitro* and *in vivo*, to reveal that Hop1 is a modular protein, consisting of a protease-sensitive N-terminal HORMA domain and a protease-resistant, C-terminal domain harbouring the Cys_2_/Cys_2_ zinc finger motif (56,61,67). During the course of these studies, we serendipitously discovered that the His_6_-tagged Hop1 expressed heterologously in *E. coli* and purified by nickel-affinity and size-exclusion chromatography to homogeneity, as evidenced by Coomassie blue staining of the SDS-gel as well as immunodetection with specific antibodies (Figure 1A), catalyzed ATP hydrolysis, generating the products ADP and inorganic phosphate (P_i_). Following this lead, we first examined the rate of [γ-^32^P]ATP hydrolysis as a function of Hop1 concentration using thin layer chromatography (TLC). Our results revealed that the intensity of P_i_ product increased as a function of various concentrations of Hop1 (Figure 1B). Quantification indicated that ∼60% of the input [γ-^32^P]ATP had been hydrolyzed by Hop1, at the highest protein concentration tested (1 μM) (Figure 1C). As such, the His_6_-tag appended to the N-terminal end of Hop1 had no discernible effect on its DNA-binding and ATPase activities.

We next sought to measure ATP binding to Hop1 using a UV cross-linking assay. To achieve this, a fixed amount of [α-^32^P]ATP was titrated against various concentrations of Hop1. In addition, *M. tuberculosis* RecA (*Mt*RecA) and *S. cerevisiae* Rev7 were used as positive and negative controls, respectively. The reaction mixtures were exposed to 365 nm UV light to induce cross-linking between ATP and Hop1. Aliquots of the reaction mixtures were spotted on a nitrocellulose membrane. Following that, membrane was washed to remove the unbound isotope and the dried membrane was exposed to a phosphorimaging plate. The results showed an increase in the intensity of radioactive species with increasing Hop1 concentrations up to 0.8 μg and then plateaued (Figure 1D and E). The reaction performed in the absence of protein showed that [α-^32^P]ATP retained on the nitrocellulose membrane was < 1% (Figure 1D, lane 2). Control reactions with *Mt*RecA showed an intense radiolabeled product, whereas *S. cerevisiae* Rev7 exhibited no signal, indicating the ATP binding specificity of Hop1.

While the foregoing results informed that Hop1 is capable of binding and hydrolyzing ATP, an alternative possibility is that the observed activity can be due to co-purification of an ATPase contaminant. To exclude this possibility, a pull-down assay was performed by incubating purified His_6_-tagged Hop1 with polyclonal anti-Hop1 antibodies. An aliquot from solubilized immune precipitate (IP) fraction was analyzed by SDS-PAGE and visualized by Coomassie blue staining. The results showed a band corresponding to Hop1 in the IP sample (Supplementary Figure S1A). In parallel, we analyzed the ATPase activity in the solubilized IP and supernatant fractions. Whilst the supernatant had residual activity, robust activity was observed in the IP fraction, indicating that the ATPase activity is associated with Hop1 protein (Supplementary Figure S1B). This was further validated by size exclusion chromatography. To this end, purified Hop1 protein was applied to a Superdex 200 gel filtration column and eluted with equilibration buffer. The recovery of Hop1 from the column was ∼94%. The ATPase activity across the elution peak was measured using a colorimetric assay (90). We found that Hop1 eluted from gel filtration column as a single peak with an apparent molecular weight of 71 kDa, coincident with a peak of ATPase activity (Supplementary Figure S1C–E). Though not definitive, this result excluded the possibility that a contaminant ATPase had co-purified with Hop1.

Although Hop1 obtained from different purification batches exhibited dose-dependent ATPase activity, we were wary of drawing the conclusion that Hop1 has intrinsic ATPase activity. To address this concern, we turned to *in vitro* transcription/translation system for cell-free synthesis of N-terminally His_6_-tagged Hop1 using rabbit reticulocyte lysates: Hop1 produced in this system was purified to homogeneity using a single Ni^2+^-NTA affinity chromatography step, as confirmed by SDS-PAGE and by staining the gel with Coomassie blue (Figure 1F, lanes 5–6). We then assessed its ability to catalyze hydrolysis of [γ-^32^P]ATP as described above. Reassuringly, we found that Hop1 produced by cell-free protein synthesis system displayed robust ATP hydrolytic activity in a concentration- and time-dependent manner (Figure 1G and H). Based on these collective results, we conclude that Hop1 has an intrinsic ATPase activity.

### Optimization of assay conditions for ATP hydrolysis by Hop1

To start characterizing the Hop1 ATPase activity, we sought to determine conditions that might enable highest ATPase activity by Hop1. The results showed that the optimal pH was between pH 6.5 and 7.5 (Supplementary Figure S2A), consistent with the intracellular pH in *S. cerevisiae* (106). The reaction accelerated when the temperature was raised from 5°C, with the optimal temperatures between 30 and 37°C and then declined: the latter might be due to thermal denaturation of Hop1 protein (Supplementary Figure S2B). Similarly, the reaction increased linearly with time and then tended to plateau at 60 min (Supplementary Figure S2C). To test whether Hop1 could hydrolyze other NTPs, we performed experiments under identical conditions and found that GTP was hydrolyzed similar to the level as seen with ATP, whereas CTP and UTP were cleaved less efficiently; but ATPγS was not cleaved at all (Supplementary Figure S2D). While studying the effect of divalent cations as cofactors, we observed unexpectedly that Hop1 exhibited robust ATPase activity in the absence of added divalent cations: this might be due to the presence of a tightly bound divalent cation. A systematic survey of literature data revealed that several enzymes catalyze reactions using bound metal ions (107–109). Interestingly, Hop1 catalyzed ATP hydrolysis to similar extents in the presence of either Mg^2+^ or Ca^2+^ ions, but decreased by 25% and 80% in the presence of Mn^2+^ and Ni^2+^, respectively, whereas both Cu^2+^ and Zn^2+^ ions inhibited ATP hydrolysis (Supplementary Figure S2E). The simplest interpretation is that Cu^2+^, Ni^2+^ and Zn^2+^ ions might inhibit ATP hydrolysis by either directly competing with Mg^2+^ or Ca^2+^ ions already bound to Hop1 or acting as negative allosteric modulators. Intrigued by the ability of Hop1 to catalyze ATP hydrolysis in the absence of an added divalent cation, we carried out experiments to test the presence of tightly bond cations using chelating agents at different concentrations. As anticipated, we found that both EDTA and EGTA inhibited Hop1 catalyzed ATP hydrolysis in a dose-dependent manner (Supplementary Figure S2F). Curiously, we noticed that addition of Zn^2+^ ion inhibited ATP hydrolysis by Hop1 (Supplementary Figure S2G), although the relevance of this effect remains unclear.

### Nucleotides induce conformational changes in Hop1

Several lines of evidence indicate that nucleotides induce conformational changes in DNA/RNA binding proteins/enzymes, which often facilitate the formation of functional complexes and their interaction with other molecules (110–113). We thus examined whether NTPs induce conformational changes in Hop1 using a thermal shift assay, with SYPRO Orange as the reporter dye, as previously described (85). The experiments were performed in reaction mixtures containing 10 μg of Hop1 in the absence or presence of 5 mM rATP, rGTP and AMP-PNP. We observed an increase in the thermal melting temperature (*T*_m_ ) of Hop1 by ∼2.0°C in the presence of ATP/GTP, whereas AMP-PNP increased by ∼1.0°C (Supplementary Figure S3A and B). These results support a model in which the extent of conformational changes depends on the nature of nucleotides bound to Hop1 protein.

### Hop1 exhibits DNA-independent ATPase activity

**Figure 2.**
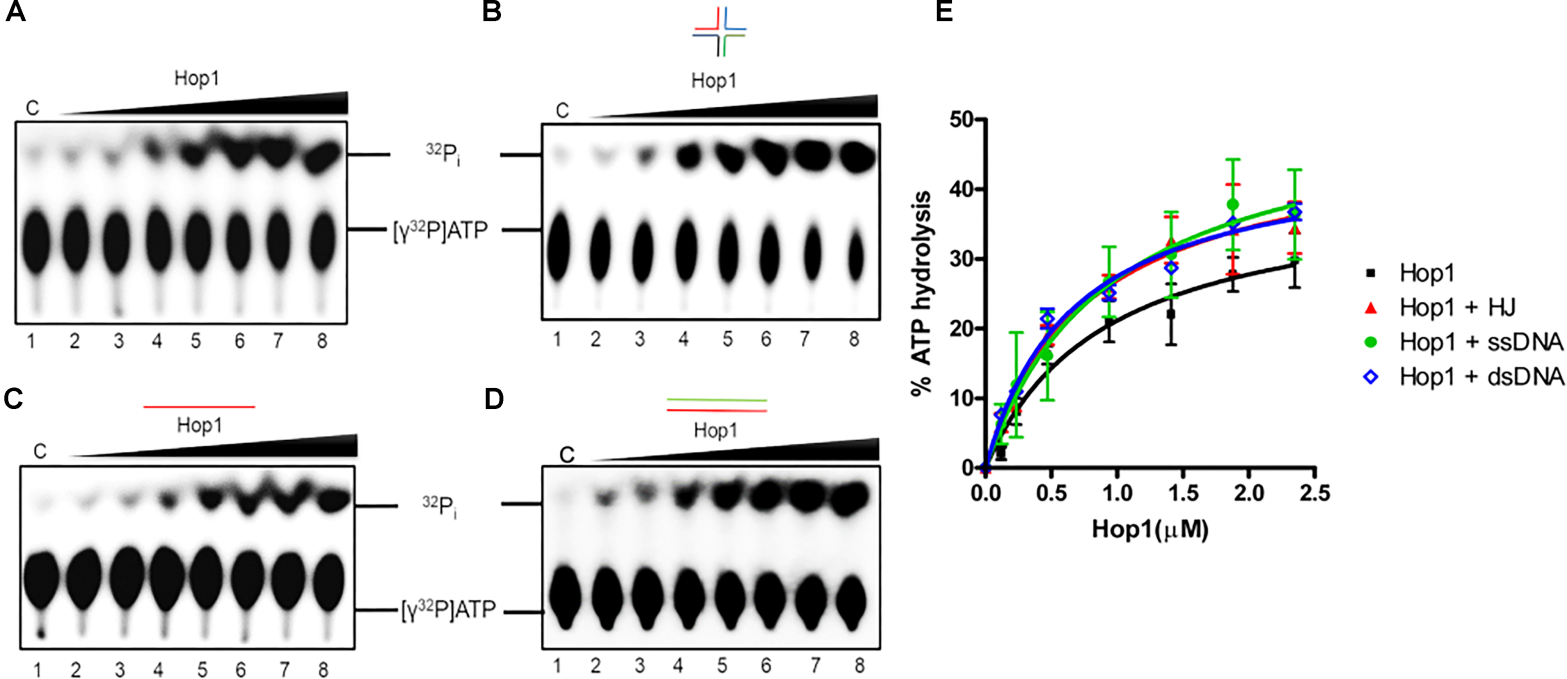
ATPase activity of Hop1 is not coupled to its DNA binding activity. (**A**) ATPase activity in the absence of DNA. (**B–D**) As in (A) but in the presence of 500 nM of the HJ, ssDNA and dsDNA, respectively. The reaction mixtures contained 50 μM cold ATP (and 200 pM [γ-^32^P]ATP as a tracer) in the absence (lane 1) or presence of 0.12, 0.23, 0.47, 0.94, 1.41, 1.88 and 2.35 μM Hop1 (lanes 2–8), respectively. (**E**) Quantitative analysis of ATP hydrolysis (mean and SD for *n* = 3) in the absence or presence of different DNA substrates as a function of various concentrations of Hop1.

Numerous studies have documented that DNA and RNA, by acting as cofactors, stimulate the NTPase activities of various enzymes that function in different pathways of nucleic acid metabolism (114, 115). Given that Hop1 binds to a variety of DNA substrates with a range of affinities (61–64), we hypothesized that DNA might modulate its ATPase activity. To address this possibility, experiments were performed in the presence of constant amounts of DNA cofactors of mixed sequence (containing all four bases) in reaction mixtures containing [γ-^32^P]ATP and varying concentrations of Hop1. The reaction products were separated by TLC and analyzed by phosphor imaging. In the absence of a DNA cofactor, the amount of [γ-^32^P]ATP hydrolyzed increased in a manner dependent on Hop1 concentration (Figure 2A and E). Similar results were obtained in the presence of three different types of DNA cofactors, namely the HJ, dsDNA and ssDNA (Figure 2B–E), suggesting that ATP hydrolysis catalyzed by Hop1 is DNA-independent. Thus, we speculate that the Hop1 ‘catalytic domain’ responsible for ATP hydrolysis might be in an active conformation. Together, our data are reminiscent of the DNA-independent ATPase activities of *M. tuberculosis* UvrC (116), *Oryza sativa* RuvBL1a (117), MukB ATPase (118,119) and *E. coli* RadB (120).

### Kinetic parameters for ATP hydrolysis by Hop1

**Figure 3.**
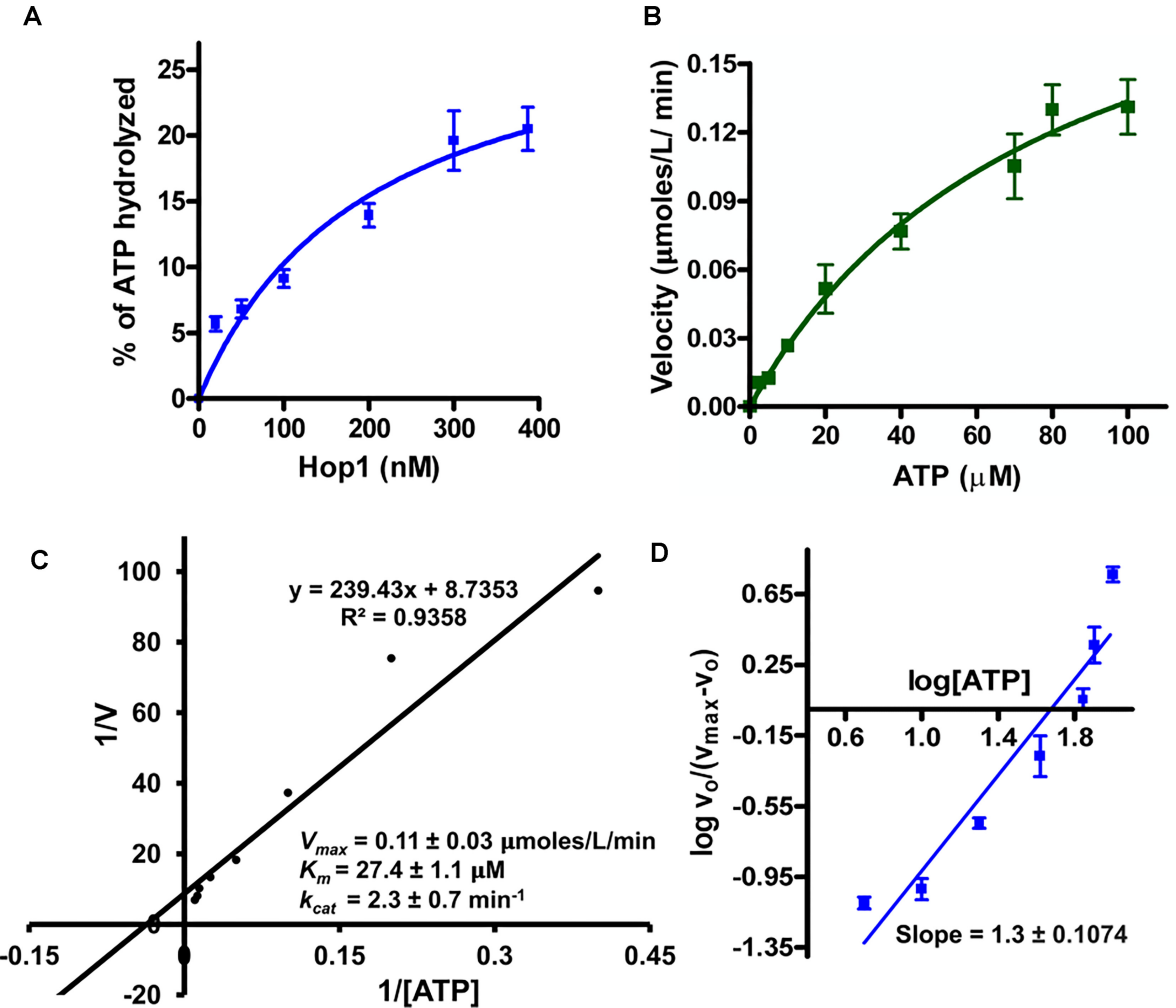
Kinetic analysis of Hop1 ATPase activity. (**A**) ATPase activity as a function of Hop1 concentration. (**B**) The reaction velocity is plotted as a function of ATP concentration. (**C**) Lineweaver–Burk plot of rate of Hop1 catalyzed ATP hydrolysis as a function of ATP. (**D**) Hill plot showing the rate of ATPase activity at different ATP concentrations. A nonlinear regression analysis was performed in panels (A) and (B) using GraphPad Prism software (version 5.0). Linear regression analysis was performed in panels (C) and (D). The error bars represent standard deviation for three independent experiments.

Building upon the aforementioned results, we sought to assess the kinetic parameters of ATP hydrolysis by Hop1. In pursuit of this objective, we determined the effect of different concentrations of Hop1 (Figure 3A) and ATP (Figure 3B) on the initial velocity of ATP hydrolysis using a sensitive colorimetric ATPase assay (90).

The results showed that the kinetics of ATP hydrolysis by Hop1 follows an apparent hyperbolic dependence on ATP concentration (Figure 3B), indicating a lack of cooperativity in ATP binding and/or hydrolysis. The concentration of Hop1 required for half-maximal saturation was ∼95 nM. Figure 3C shows a Lineweaver–Burk plot of ATP hydrolysis by Hop1, as a function of the ATP concentration. Fitting the kinetic data to the Michaelis–Menten equation revealed apparent *K*_m_(ATP) and *V*_max_ values of 27.4 ± 1.11 μM and 0.11 ± 0.03 μmoles L^−1^min^−1^, respectively. Furthermore, the catalytic rate constant (*k*_cat_) and catalytic efficiency (*k*_cat_*/K*_m_) were 2.3 ± 0.7 min^−1^ and 0.082 ± 0.001 min^−1^ μM^−1^, respectively. The Hill coefficient was 1.3 ± 0.10, which is indicative of a single binding site on Hop1 for ATP (Figure 3D).

### Hop1 lacks recognizable motifs needed for ATP binding and hydrolysis

To identify the motifs implicated in ATP-binding and/or hydrolysis, the deduced amino acid sequence of Hop1 was aligned against amino acid sequences of ATP-binding and hydrolysis motifs using Clustal Omega webserver (https://www.ebi.ac.uk/jdispatcher/msa/clustalo). This bioinformatic query, to our surprise, revealed that Hop1 has no sequence similarity with ATPases containing the Walker A motif, P-loop-associated motif, Q-motif, DEAD box motif and C-loop motif (Supplementary Figure S4).

### Computational tools predict the existence of a putative ATP-binding site in Hop1

As Hop1 lacks recognizable sequence motifs required for ATP-binding and/or hydrolysis, *in silico* methods were leveraged to construct molecular models of Hop1 in complex with ATP in order to identify aa residues that are potentially involved in ATP binding and/or hydrolysis. For this purpose, Hop1 primary sequence was submitted to the I-TASSER pipeline for automated full-length protein structure and function prediction (121–123). Interestingly, this approach suggested that Hop1 could interact with a family of ligands, including ATP, GTP, ADP and AMP-PNP (124). The gene ontology (GO) analysis showed that Hop1 belongs to the family of proteins that bind to adenyl ribonucleotides (GO:0032559) or function as triphosphatases (GO:0016462)/pyrophosphatases (GO:0035639) (Supplementary Figure S5). Due to the overall high degree of sequence divergence of HORMA domain, a partial structure was modeled, which had low confidence scores. Therefore, we sought to generate a high confidence structure of Hop1 using a combination of multi-template modeling and molecular threading.

**Figure 4.**
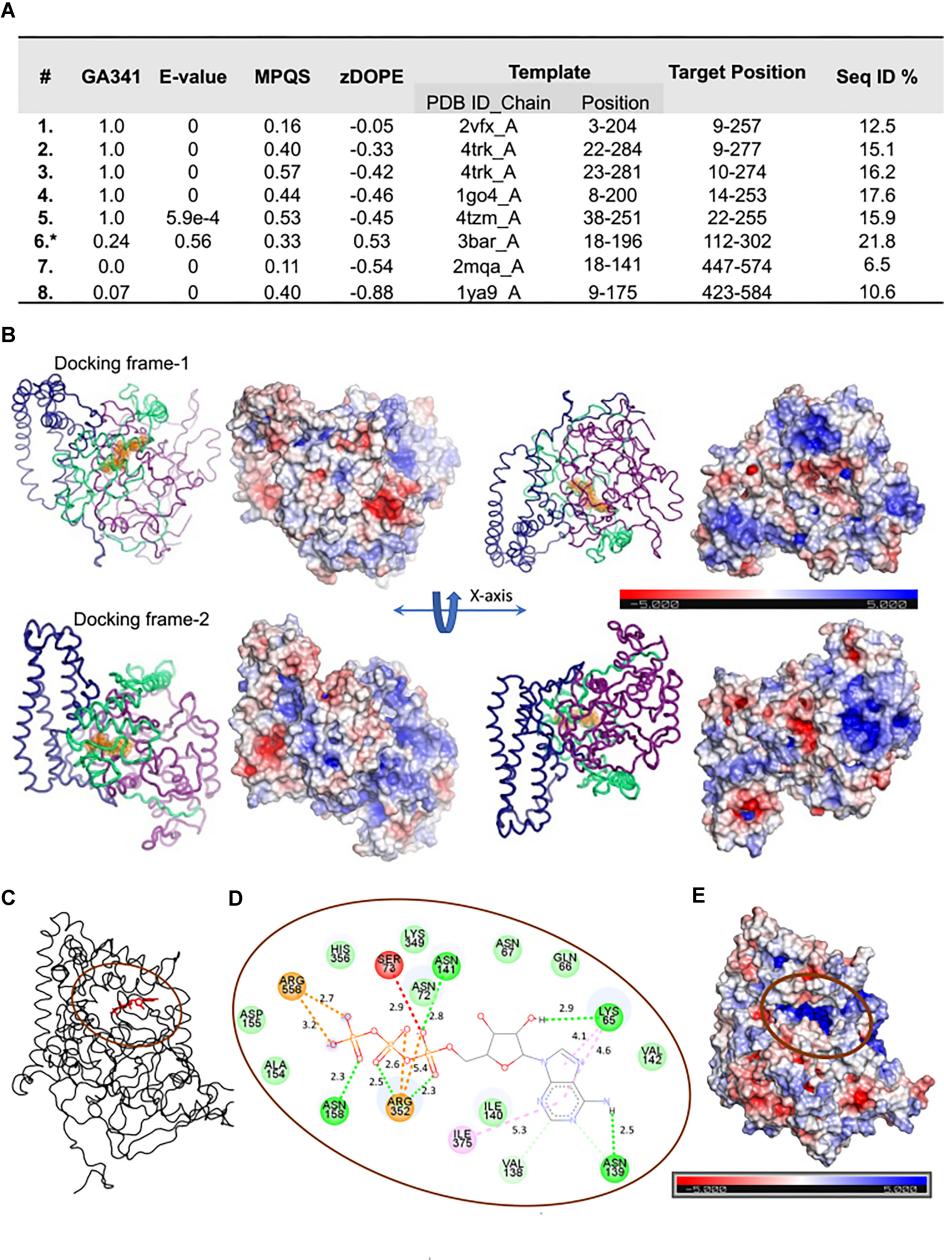
*In silico* prediction algorithms, molecular docking and MD simulations reveal a putative ATP-binding site in Hop1. (**A**) Quality statistics of template fragments used to construct the Hop1 model. The 6 with an asterisk (*) corresponds to a template with a poor match, but it is included for guiding the modeling of the extended loop regions. (**B**) Cartoon diagram showing two Hop1 models (frame 1 and frame 2) selected from all-atom MD simulation for docking ATP. The segments 1–277, 278–422 and 423–605 are marked in deep purple, forest green and deep blue, respectively. The electrostatic surface is shown adjacent to models in the same orientation for comparison. The protein view is shown from both faces after rotation around the *X*-axis by 180°. The position of the top-ranked docked-ATP ligand is shown as orange spheres. Note that although the docking modes of ATP in frame 1 and 2 differ, they remain adjacent. (**C**) Docking simulations of the Hop1-ATP complex (frame 1). The location of the bound ATP after 200 ns MD simulation. (**D**) Schematic diagram showing the ATP binding site and various interactions (dashed lines) between ATP and Hop1. The oval indicates the primary region of interest. The acceptor atoms in the ATP molecule are indicated, and the distance in Å is marked for each dashed line between a donor–acceptor. In regard to a stacking interaction, the dashed line points to the corresponding location. (**E**) The surface view of the docking orifice of the ATP ligand. The electrostatic surface potential at the docking site is colored deep blue. Panels (C) and (E) are drawn in the same orientation. The electrostatic surface potential was calculated using the APBS plugin within the PyMol software (https://pymol.org), which was also used to produce all cartoon diagrams of the structure.

The models built using the multi-template modeling show a compact structure wherein the N-terminal and C-terminal segments are bound to each other, forming a groove-like structure with the peptide linker containing the zinc finger motif. GA341 denotes the model score derived from statistical potentials, wherein a value >0.7 is indicative of a reliable model that exhibits ≥95% probability of correct protein folding. The *E*-value represents the significance of the target-template sequence alignment computed after template search, wherein the values close to zero suggest reliability. The ModPipe Quality Score (MPQS) signifies a composite model score based on sequence identity, *E*-value, template coverage, GA341 and zDOPE. An MPQS value >1.1 generally indicates a reliable model (Figure 4A). As noted above, Hop1 is a modular protein, consisting of an N-terminal HORMA domain and a protease-resistant C-terminal domain (61). A close inspection of our structures revealed that large parts of the protein contains a positively charged surface, consistent with the observations that Hop1 interacts via the negatively charged phosphate backbone of DNA (62,64). The zinc finger motif located between the Hop1’s N- and C-terminal domains plays a vital role in stabilizing the tertiary structure (39,61). These findings provided great confidence in the predicted model, with 1.8% of the main-chain torsion angles in the disallowed region of the Ramachandran plot, 5.9% in the generously allowed region and the remaining 92.3% in the allowed region. Further, the WHAT_CHECK structure validation program (125) indicated no significant errors in the structural model.

### Identification of amino acid residues in the Hop1 ATP-binding site that interact with ATP

The MD simulations were carried out to explore the interaction between ATP and Hop1. Interestingly, MD simulations showed that Hop1 interacts with phosphate moieties of bound ATP. The frames 1 and 2 in Figure 4B were found to be the top ranked structures wherein ATP docked at two adjacent locations with energy scores of −9.2 and −9.0 kcal/mole, respectively. For frame 1, there was another hit at the second rank at the same site, whereas for frame 2, there were two other *in silico* docking hits at the same site. Considering all the hits, the nearest distance between the bound ATPs at the two sites is 3.5 Å. Essentially, the docking in both the frames happened through the same surface orifice, where the ATPs docked shallow versus deep in frames 1 and 2, respectively. The lower-ranked ATP hits showed similar docking at the two frames. Therefore, given that the top frame 1 hit had a better affinity score and was comparatively closer to the surface, we considered it important enough for further analysis. Our results pin point that ATP is positioned in a deep cleft (Figure 4C, oval symbol), bound by hydrophilic residues such as Arg, Asp, Asn, Gln, His and Lys (Figure 4D), creating a highly positively charged docking site (Figure 4E). Although the region between the N- and C-terminal regions contributes to the ATP-binding surface, residues in the N-terminal HORMA regions (K65, N67 and N139) and the C-terminal region (R352 and R558) interact with the ATP molecule (Figure 4D). Furthermore, the stability of the Hop1 structure seemed to increase after *in silico* docking of ATP. Additionally, the distribution of positively charged and hydrophobic residues in the site is complementary to the location of the phosphate and the base region of ATP, making this site a particularly strong candidate for interaction with ATP.

### The Hop1 putative ATP-binding site is fairly conserved across different species from diverse fungal lineages

To test whether the residues that form the Hop1 ATP‐binding site are conserved, the amino acid sequences of *S. cerevisiae* Hop1 were aligned with orthologs from representative fungal species, such as *Fabospora*, *Torulaspora*, *Candida*, *Kluyveromyces* and *Naumovozyma* using the PSI-BLAST program. Gratifyingly, we observed that K65 is remarkably well-conserved, whereas R352 and R558 (substituted by lysine) are fairly conserved and N67 and N139 are less conserved (Supplementary Figure S6). These data suggest that the putative ATP binding/hydrolysis site of Hop1 is generally conserved among representative species derived from diverse fungal lineages.

### Purification and characterization of WT Hop1 and its variants

**Figure 5.**
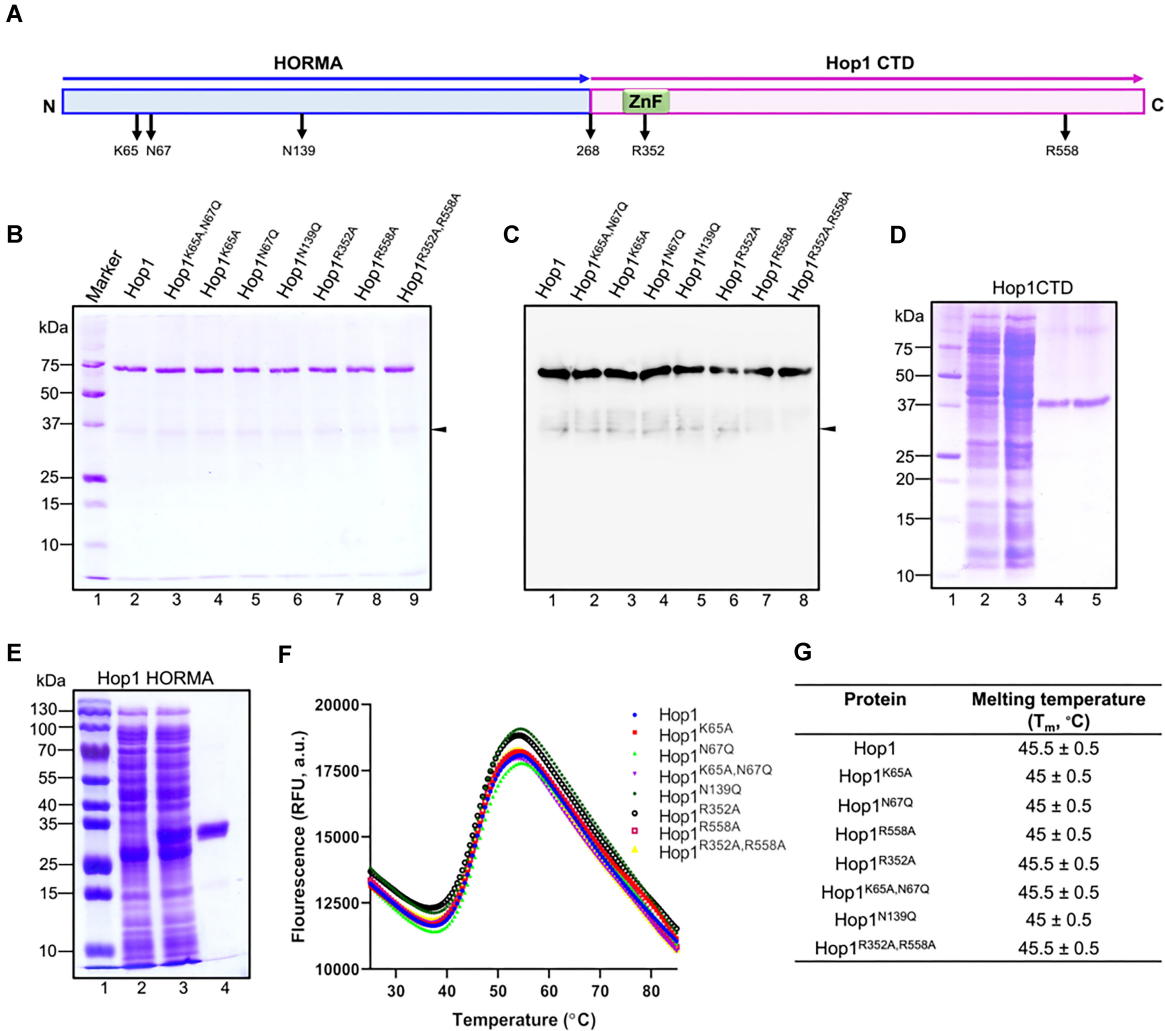
Purification, SDS-PAGE analysis and thermal stability of WT Hop1 and its variants carrying amino acid substitutions in the putative ATP binding site. (**A**) Schematic diagram of Hop1 domain structure: an N-terminal HORMA domain, and a C-terminal domain (Hop1-CTD) including the zinc finger motif. The aa residues potentially involved in ATP binding/hydrolysis are indicated below the linear schematic diagram. (**B**) Shown is a Coomassie blue-stained SDS-PAGE gel showing the homogeneity of purified WT and Hop1 variants. Lane 1, molecular weight standards. Lanes 2–9, SDS-PAGE analysis of purified WT Hop1 and its variants (5 μg protein in each lane). (**C**) Western blot analysis of WT Hop1 and its variants using anti-Hop1 antibodies. The closed arrowheads in panels (B) and (C) denote the Hop1 degradation product. (**D**) A Coomassie blue-stained SDS-PAGE gel of protein samples from various stages of Hop1-CTD purification. Lane 1, molecular weight standards; lane 2, whole-cell lysate from uninduced cells (25 μg protein); lane 3, whole-cell lysate from induced cells (25 μg protein); lane 4, eluate from Ni^2+^-NTA affinity column (4 μg protein); lane 5, eluate from Superdex 200 column (4 μg protein). (**E**) A Coomassie blue-stained SDS-PAGE gel of protein samples from various stages of purification of Hop1 HORMA domain. Lane 1, molecular weight standards; lane 2, whole-cell lysate from uninduced cells (30 μg protein); lane 3, whole-cell lysate from induced cells (30 μg protein); lane 4, eluate from Ni^2+^-NTA affinity column (5 μg). (**F**) Thermal denaturation profiling of WT Hop1 and its variants. (**G**) *T*_m_ values of WT Hop1 and its variants. The data are presented as the mean ± SD from three independent experiments.

The molecular docking of ATP and MD simulations revealed that an ensemble of five hydrophilic residues, namely, K65, N67, N139, R352 and R558, in Hop1 directly interact with ATP (Figures 4D and 5A). To experimentally validate the *in silico* predictions, five single (K65A, N67Q, R352A, R558A and N139Q) and two double (K65A,N67Q and R352A,R558A) substitutions were introduced in the putative ATP-binding site of Hop1 via site-directed mutagenesis as previously described (126). The WT Hop1 and its variants were expressed in *E. coli* and purified to > 95% homogeneity, as evidenced by Coomassie blue staining of the SDS-gel as well as western blot analysis. Hop1 variants were purified using the same method as for the WT species with comparable yield and purity (Figure 5B and C). Similarly, the N- and C-terminally truncated variants of Hop1 were expressed in *E. coli* and purified to homogeneity (Figure 5D and E) as previously described (61). Differential scanning fluorimetry (DSF) has been widely used to assess protein thermal stability induced by mutations and orthologous proteins from different species (91,92). Using DSF, with SYPRO orange as the reporter dye, we examined the effect of amino acid substitutions on the thermal stability of Hop1 variants over a wide temperature range of 25–95°C. Interestingly, we found that the *T*_m_ values of various Hop1 variants were similar to the WT species (Figure 5F and G). Furthermore, the WT Hop1 and its variants analyzed here displayed a similar behavior during gel filtration chromatography and SDS-PAGE. In summary, these results suggest that amino acid substitutions in the putative ATP-binding site do not appear to disrupt the three-dimensional structures that determine the stability of Hop1 variants.

In the next two sections, we describe the impact of substitution of aa residues in the ATP-binding pocket on ATP-binding and hydrolysis activities of Hop1.

### Hop1 binds ATP with high affinity

**Figure 6.**
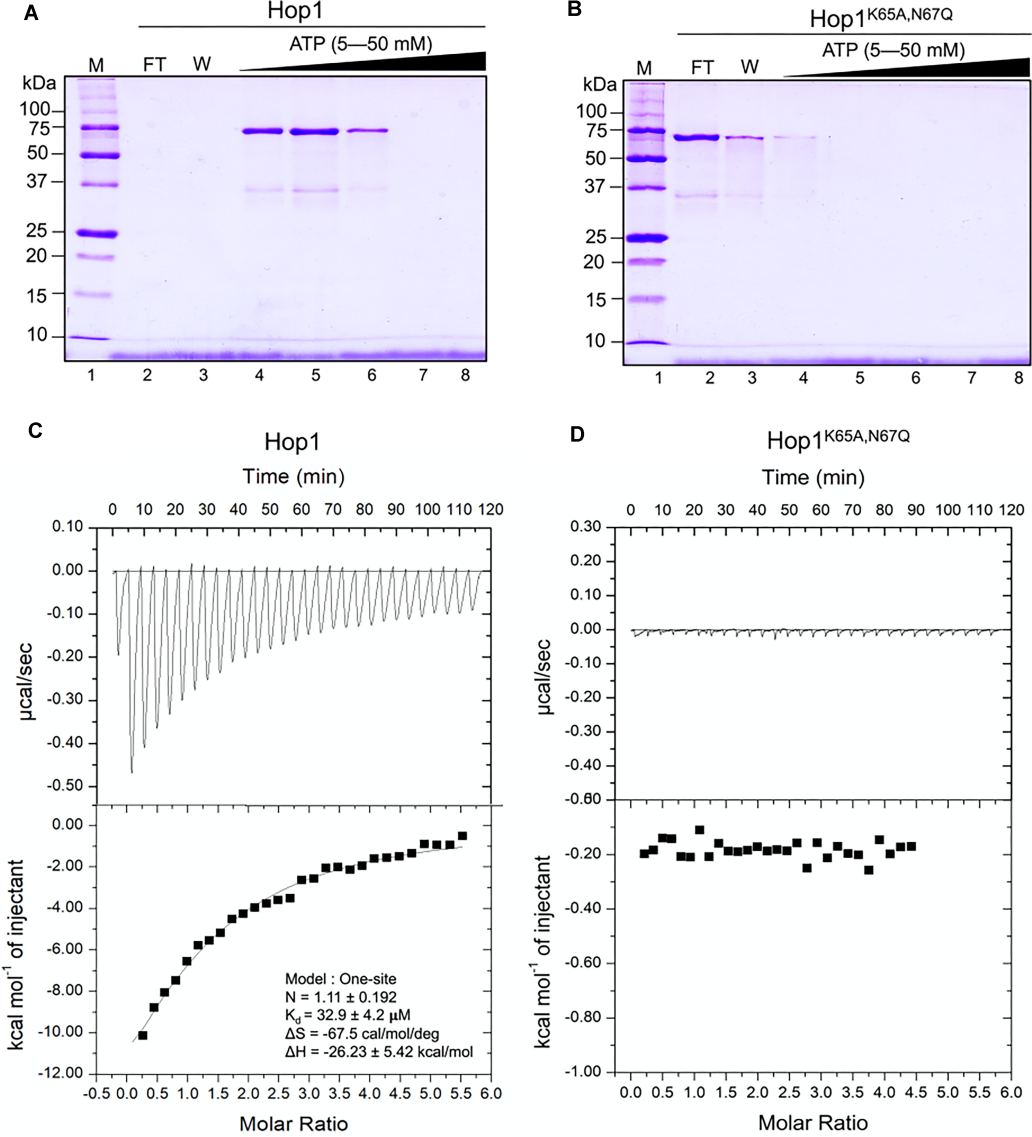
Wild-type Hop1, but not its Hop1^K65A,N67Q^ variant, binds ATP. (**A**) WT Hop1 bound to ATP-agarose resin could be eluted with ATP. (**B**) The Hop1^K65A,N67Q^ variant does not bind to ATP-agarose. (A and B) The indicated samples were analyzed by SDS-PAGE and visualized by staining with Coomassie blue. Lane 1 (M), standard molecular markers; lane 2 (FT), flow-through; lane 3 (W), wash fraction; lanes 4–8, fractions eluted with 5, 10, 20, 30 and 50 mM ATP, respectively. (**C**) A representative ITC thermogram of the WT Hop1-ATP complex formation; heats of injection are shown on the top panel. The thermodynamic parameters obtained are indicated in the inset. (**D**) A representative ITC thermogram of the complex formation between Hop1^K65A,N67Q^ variant and ATP; heats of injection are shown on the top panel. All injections were performed at 240 s intervals. The experiments were performed as described in the ‘Materials and methods’ section. Data shown are representative of two independent titrations.

We reasoned that insights into the potential ATPase activity of Hop1 might be revealed by analyzing the interaction of Hop1 with ATP in more detail. To test this premise, samples of Hop1 WT and Hop1^K65A,N67Q^ variant were chromatographed on ATP-agarose. After washing the matrix extensively, the bound proteins were eluted using various concentrations of ATP. The eluted fractions were analyzed by SDS-PAGE and visualized by Coomassie blue staining. The results showed that the WT species remained bound to the ATP-agarose even after extensive washing, whereas the Hop1^K65A,N67Q^ variant was recovered in the flow-through and wash fractions (Figure 6A and B). The bound WT species was eluted using a linear gradient ranging from 5 to 50 mM ATP (Figure 6A). As previously described (61), the faster migrating species seen in Figure 6A (lanes 4–6) correspond to the autoproteolytic cleavage product of full-length Hop1.

Inspired by the above results, we carried out isothermal titration calorimetry (ITC) measurements to determine the thermodynamic parameters (Δ*S* and Δ*G*) along with the binding affinities (*K*_d_ values) for the interaction of ATP with WT Hop1 and Hop1^K65A,N67Q^ variant. The titrations of WT Hop1 with ATP led to upward shift of the ITC signals upon each injection until the signal has reached a steady state (Figure 6C). The kinetic data revealed a *K*_d_ of ∼32.9 ± 4.2 μM (Figure 6C). The thermodynamic parameters are shown in the inset of Figure 6C (bottom panel). A similar titration with the Hop1^K65A,N67Q^ variant showed no ITC signals upon addition of ATP (Figure 6D), suggesting that its binding capacity was compromised, which is consistent with the aforementioned findings. Together, our data and analysis indicate that K65A and N67Q substitutions in the ATP binding site completely abrogated the ability of Hop1^K65A,N67Q^ variant to bind ATP.

### Amino acid substitutions in the putative ATP-binding site differentially impair Hop1 ATPase activity

**Figure 7.**
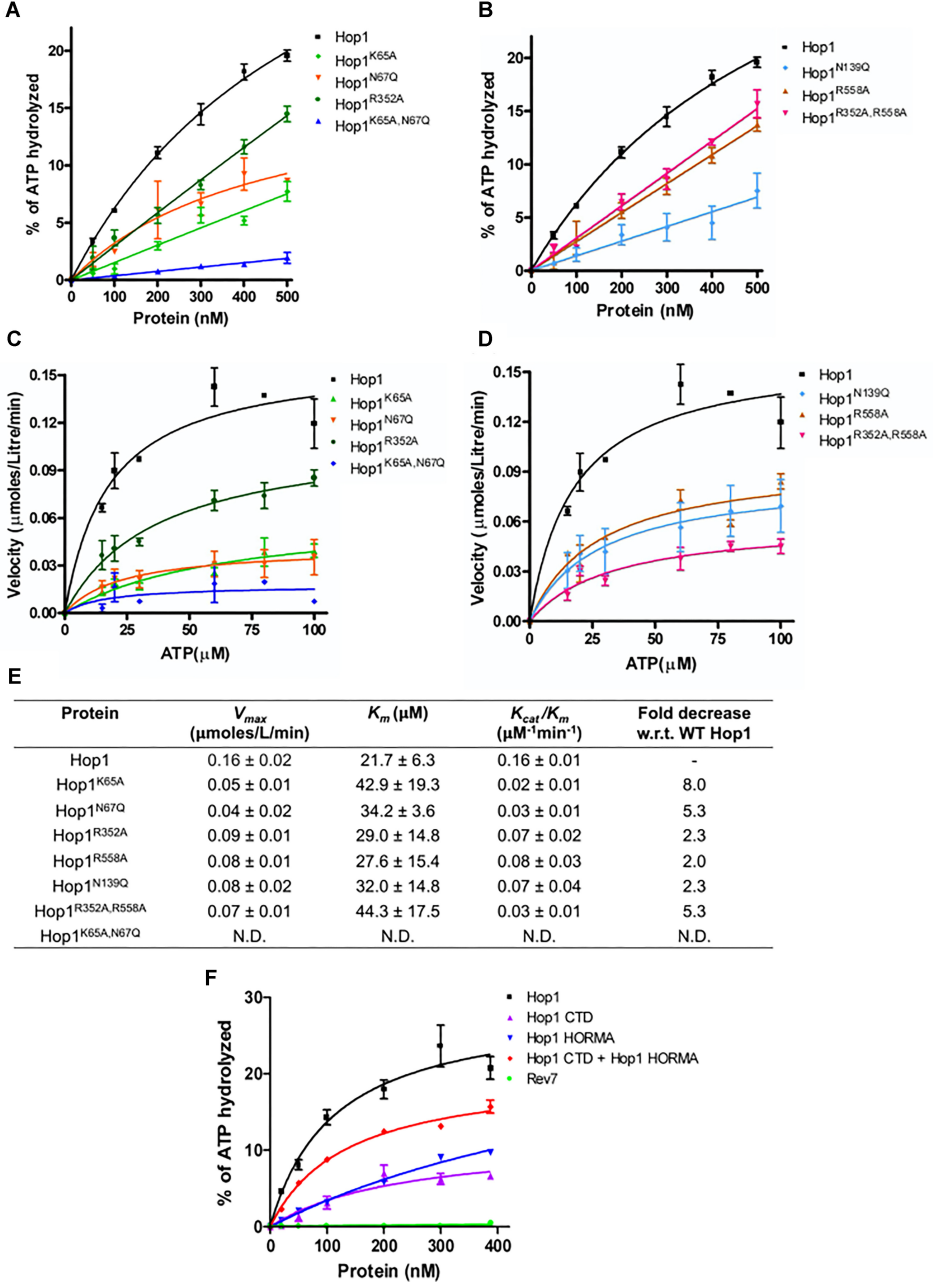
Amino acid substitutions in the putative ATP binding site of Hop1 impair ATP hydrolysis to varying degrees. (**A** and **B**) ATPase activity of purified WT Hop1 and its variants was determined as a function of protein concentration. Various concentrations (50, 100, 200, 300, 400 and 500 nM) of WT Hop1 and its variants were individually incubated with 100 μM of ATP at 37°C for 80 min. (**C** and **D**) The rate of ATP hydrolysis WT Hop1 and its variants (50 nM) as a function of ATP concentration (0, 15, 20, 30, 60, 80 and 100 μM ATP). The data presented for WT Hop1 in (C) are reused in (D). (**E**) The kinetic parameters for ATP hydrolysis by WT Hop1 and its variants. (**F**) Mixing of the N- and C-terminal domains of Hop1 partially restored ATPase activity. The assay was performed as in (A), but in the absence or presence of 20, 50, 100, 200, 300 and 387 nM of WT Hop1, or equal concentrations of N- and C-terminal domains of Hop1. *S. cerevisiae* Rev7 was used as a negative control at the same concentration. The data were assessed using nonlinear regression analysis in GraphPad Prism (version 5.0 software). Error bars represent standard deviation across three independent experiments.

Next, we sought to test whether amino acid substitutions in the Hop1’s ATP-binding site (Figure 4D) impact its ATPase activity. To do this, kinetic experiments were carried out using purified WT Hop1 and its variants over a range of protein and ATP concentrations (Figure 7A–D) using a colorimetric assay (90). The results showed that ATP hydrolysis catalyzed by WT Hop1 and its variants, except the Hop1^K65A,N67Q^ variant, was directly proportional to the enzyme concentration (Figure 7A and B). Furthermore, the rate of ATP hydrolysis increased with increasing ATP concentrations up to 60 μM and plateaued thereafter (Figure 7C and D). The estimated values of *K*_m_ and *V*_max_ for Hop1 WT were 21.7 ± 6.3 μM and 0.16 ± 0.02 μmoles L^−1^ min^−1^, respectively. Interestingly, amino acid substitutions in the Hop1 ATP binding site reduced the ATPase activity of Hop1 variants, albeit to varying extents (Figure 7C and D). While the catalytic efficiency (*k*_cat_/*K*_m_) of R352A and R558A variants was reduced by ∼2-fold, a 5-fold decrease was seen with the N67Q and K65A,N67Q variants (Figure 7E). On the other hand, the K65A variant displayed an 8-fold reduction in its catalytic efficiency, but K65A and N67Q substitutions synergistically abolished ATPase activity (Figure 7E). These findings, consistent with *in silico* molecular docking (Figure 4D) and ATP-binding data (Figure 6D), suggest that residues K65 and N67 are necessary for Hop1 to bind ATP and catalyze its hydrolysis.

### The residues in both the N- and C-terminal regions are required for Hop1 ATPase activity

As previously noted, Hop1 possesses a modular structure, consisting of an N-terminal HORMA region and a C-terminal terminal region; the latter is indispensable for its DNA binding activity (61). Encouraged by the importance of residues K65 and N67 for the ATPase activity of Hop1, we considered whether the N-terminal domain alone is enough to display WT levels of ATPase activity. Our results showed that its ATPase activity was ∼50% of the full-length Hop1 (Figure 7F), suggesting that N-terminal domain is necessary but not sufficient. We then interrogated the ability of Hop1-CTD to catalyze ATP hydrolysis under similar conditions and found that it promotes ATP hydrolysis at levels similar to that of the N-terminal region. Given the *in silico* data (Figure 4), we reasoned that residues in both N- and C-terminal regions may be required for Hop1 mediated ATP hydrolysis. Remarkably, mixing equal amounts of the N- and C-terminal regions partially restored the ATPase activity (Figure 7F), suggesting that functional interaction between residues in the N- and C-terminal regions of Hop1 were necessary for its ATPase activity. To validate this premise, direct interaction between the N- and C-terminal regions was tested using MST and the far-western blot analyses. The results revealed robust interaction between the N- and C-terminal regions of Hop1 with an apparent *K*_d_ value of 1.9 ± 0.5 μM (Supplementary Figure S7A–C). These findings provide a possible explanation for the lack of consensus sequence motif for ATP/hydrolysis in Hop1. As anticipated, we found that the *S. cerevisiae* Rev7, a founding member of the HORMA domain-containing proteins, showed no ATP hydrolysis activity.

### Substitutions of amino acids in the ATP-binding site impact the HJ binding affinity of Hop1

**Figure 8.**
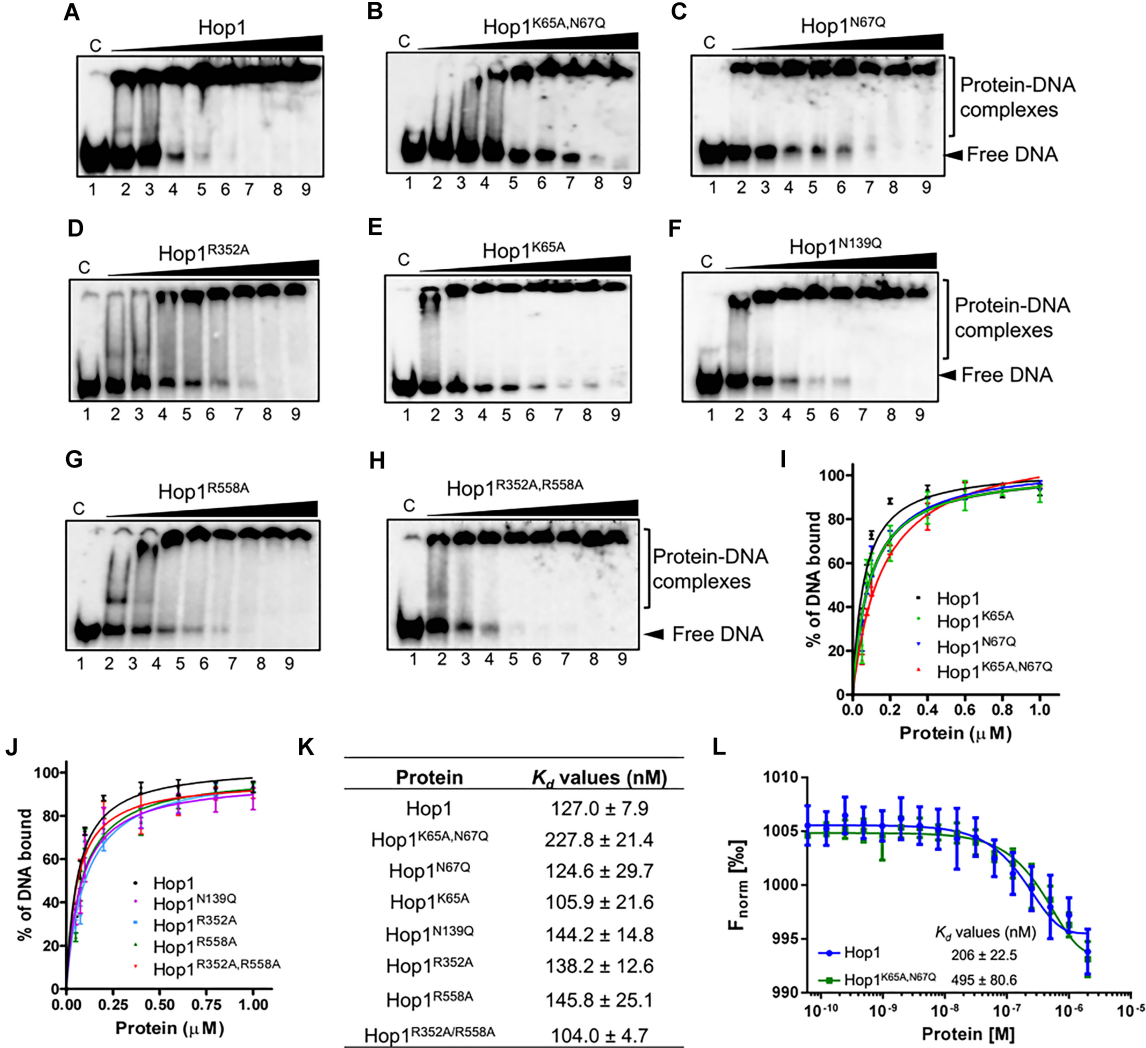
The K65A/N67Q substitutions reduce the HJ binding affinity of Hop1. (**A**) A representative gel image showing the HJ binding activity of WT Hop1. Lane 1, HJ alone. Lanes 2–9, reactions performed with 0.5 nM of ^32^P-labeled HJ and various concentrations of WT Hop1 (0.05, 0.075, 0.1, 0.2, 0.4, 0.6, 0.8 and 1 μM). (**B–H**) Representative gel images from PAGE analysis demonstrating the HJ-binding activities of different variants of Hop1. Experiments were done as discussed above, but with variant Hop1 proteins. In (A–H) C denotes the reference control. The filled triangle on the top of each gel image indicates various concentrations of WT Hop1 or its variants. (**I**and**J**) Quantitative analysis of EMSA results.The data are from three independent experiments. The data presented for WT Hop1 in (I) is reused in (J). (**K**) The apparent *K*_d_ values for interaction of WT Hop1 and its variants with the HJ. (**L**) MST isotherms for the binding of WT Hop1 and Hop1^K65A,N67Q^ variant to the 5′-6-FAM labeled HJ. The ± signs shown represent standard error of the mean (SEM). The data were analyzed by nonlinear regression using GraphPad Prism software (version 5.0 software). Error bars represent standard deviation (*n* = 3).

We showed previously that Hop1 binds to the HJ with remarkably high affinity (with a *K*_d_ of ∼32 nM) and specificity among the various types of DNA substrates tested *in vitro* (61,64). Thus, by leveraging electrophoretic mobility shift assay (EMSA), we compared the HJ binding activities of ATPase-deficient Hop1 variants with that of the WT species. We typically performed titrations for the WT Hop1 or its variants against a fixed concentration of the ^32^P-labeled HJ substrate. The results showed that the HJ-binding capacity of Hop1 variants with single amino acid substitutions in the ATP-binding site was largely similar to the WT Hop1 (compare Figure 8A with Figure 8C–H). Of note, the Hop1^K65A,N67Q^ variant showed impaired affinity for the HJ compared with the WT Hop1 (Figure 8B). Quantification of band intensities corresponding to protein–DNA complexes in each gel lane confirmed that the amount of HJ bound increased as a function of various concentrations of Hop1 or its variants (Figure 8I and J).

We further measured the HJ binding affinity (*K*_d_) of WT Hop1 and its ATP hydrolysis deficient variants using EMSA. The results revealed that, while the binding affinity of single substitutions in the ATP binding site were very similar to those of WT, the affinity of the Hop1^K65A,N67Q^ variant was ∼1.7-fold less compared to the WT species (Figure 8K). To enable accurate quantification of binding affinity, we turned to MST, which allows quantitative analysis of molecular interactions in solution when a temperature gradient is applied (127). While maintaining the concentration of 5′-6-FAM labeled HJ at 25 nM, WT Hop1 and the Hop1^K65A,N67Q^ variant were individually titrated into the reactions, and the change in DNA fluorescence was recorded. The results showed that Hop1^K65A,N67Q^ variant has a reduced HJ binding affinity. Global fitting of the binding isotherms of the WT Hop1 and Hop1^K65A,N67Q^ variant to the HJ yielded *K*_d_ values of 0.206 and 0.495 μM, respectively (Figure 8L). Together, our data and analysis indicate that the affinity of Hop1^K65A,N67Q^ variant for the HJ was reduced by ∼2.5-fold relative to the WT species. Based on these results, we chose Hop1^K65A,N67Q^ variant for interrogating the importance of Hop1’s ATPase activity during meiosis.

### Substitutions of K65A and N67Q in the ATP-binding site did not impede meiotic progression and spore formation

**Figure 9.**
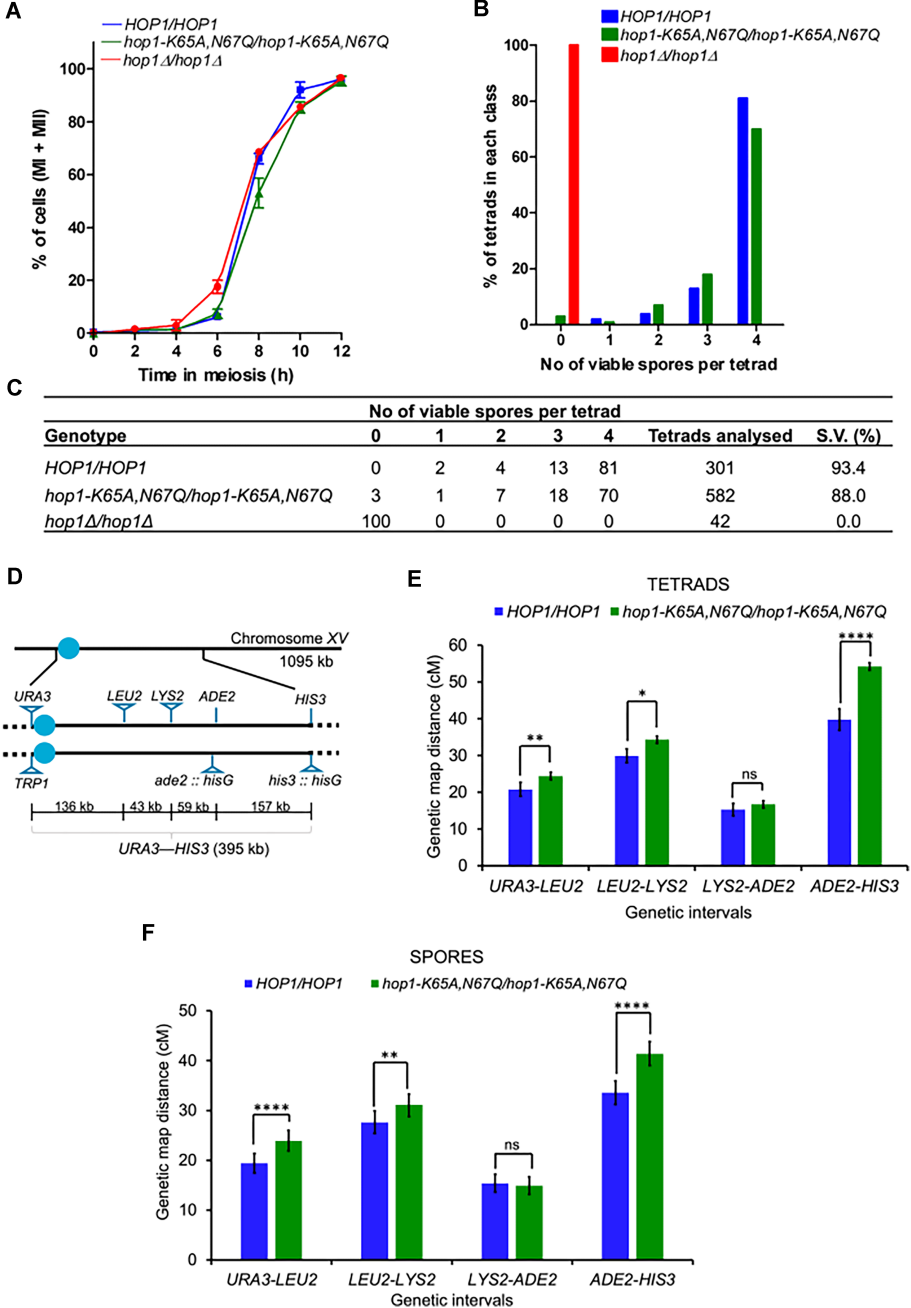
Analysis of meiotic progression, spore viability and meiotic CO frequency in the WT and *hop1-K65A,N67Q/hop1-K65A,N67Q* strains. (**A**) The percentage of cells going through meiosis I and meiosis II at the indicated time points in *HOP1/HOP1*, *hop1Δ/hop1Δ* and *hop1-K65A,N67Q/hop1-K65A,N67Q* strains. Approximately 100 cells per group were analyzed by DAPI staining. (**B**) Spore viability of WT, *hop1-K65A,N67Q/hop1-K65A,N67Q* and *hop1Δ/hop1Δ* strains. (**C**) Summary of number of tetrads analyzed and percentage of spore viability. (**D**) Schematic diagram showing genetic markers and physical distances in the *URA3-HIS3* interval on chromosome XV (redrawn from (100)). (**E** and **F**) The bar plots show genetic distances in four adjacent intervals (*URA3-LEU2*, *LEU2-LYS2*, *LYS2-ADE2* and *ADE2-HIS3*) on chromosome XV obtained upon analysis of tetrads (E) and spores (F). The error bars indicate standard error and 95% confidence interval in panels. The G-test spread sheet obtained from the Handbook of Biological Statistics (https://www.biostathandbook.com/) was used to perform the statistical analyses. ****P < 0.0001; **P< 0.01, *P< 0.05, and ‘ns’ indicates not significant. Raw data are presented in the Supplementary Table S6A and B.

What could be the biological significance of the Hop1’s ATPase activity? In most organisms, the progression of meiosis is delayed in response to defects in DSB repair and chromosome synapsis (128). Given that Hop1 plays multiple roles during meiotic prophase I (19,28,29,45,129,130), we hypothesized that the loss of ATPase activity might impact the dynamics of meiotic progression and spore formation. To test this hypothesis, the meiotic phenotype of *hop1-K65A,N67Q/hop1-K65A,N67Q* strain was compared with those of the *HOP1/HOP1* and *hop1Δ/hop1Δ* strains as previously described (131). The results showed that the percentage of *hop1-K65A,N67Q/hop1-K65A,N67Q* cells that had undergone meiosis I and meiosis II divisions was equivalent to those of the *HOP1/HOP1* and *hop1Δ/hop1Δ* strains (Figure 9A). Together, our data and analysis indicate that the absence of ATPase activity of Hop1 has no significant effect either on meiotic progression or spore viability.

### Loss of Hop1 ATPase activity leads to an enhancement in meiotic CO frequency

In most organisms, the number and distribution of meiotic COs are highly regulated (3,4). In *S. cerevisiae*, CO assurance and CO interference during meiosis are positively regulated by the ZMM group of proteins (comprising of Zip1–4, Msh4–5, Mer3 and Spo16) by antagonizing the activity of STR (Sgs1–Top3–Rmi1) complex (45,132,133). Since K65A and N67Q substitutions completely abolished the ATPase activity of Hop1^K65A,N67Q^ variant and reduced its affinity for the HJ, we asked whether these substitutions impact the meiotic COs and gene conversion in the *hop1-K65A,N67Q/hop1-K65A,N67Q* strain. To assess this, we first compared the viability of spores produced by the *hop1-K65A,N67Q/hop1-K65A,N67Q*, *hop1Δ/hop1Δ* with the WT strain. Parenthetically, these strains are derived from the sporulation-proficient SK1 strains EAY1108 and EAY1112 (100). The results informed that the spore viability of the *hop1-K65A,N67Q/hop1-K65A,N67Q* strain was comparable to the WT strain (Figure 9B and C). As previously observed (19), the spores produced by the *hop1Δ/hop1Δ* strain were 100% inviable (Figure 9B and C).

We then estimated the map distances across four intervals containing auxotrophic markers on chromosome XV in the *hop1-K65A,N67Q/hop1-K65A,N67Q* strain relative to the WT strain (Figure 9D). We found that the mutant (129.6 cM) exhibited an overall increase in the map distance of 1.22-fold in the *URA3-HIS3* interval relative to the WT strain (105.8 cM) as assessed from tetrads (Figure 9E and Supplementary Table S6A). Similarly, the overall map distances from single spore data for the *hop1-K65A,N67Q/hop1-K65A,N67Q* strain (111.3 cM) was 1.15-fold higher than the WT strain (96 cM) (Figure 9F and Supplementary Table S6B), revealing that ATP hydrolysis by Hop1 impacts the CO events during meiosis. The enhancements in map distances were significant at specific genetic intervals. For both tetrad and spore data, *URA3-LEU2, LEU2-LYS2* and *ADE2-HIS3* intervals showed a statistically significant increase in map distance for the *hop1-K65A,N67Q/hop1-K65A,N67Q* strain compared with the WT (Figure 9E and F). This is very similar to the *pch2Δ* mutant, which exhibited a 1.5-fold increase in CO rates (134,135). Further, the aberrant segregation events, involving auxotrophic markers, displayed by the *hop1-K65A,N67Q/hop1-K65A,N67Q* strain was similar to the WT cells (Supplementary Table S7). Collectively, these results support the notion that the ATPase activity of Hop1 subtly influences the CO recombination outcomes.

The positioning of COs along chromosomes is not random: Interference ensures that COs are evenly spaced by inhibiting closely spaced COs (136). Given that the CO frequency showed a modest but significant increase in the *hop1-K65A,N67Q/hop1-K65A,N67Q* strain, we calculated the coefficient of coincidence (CoC), which is the ratio of observed double COs to expected double COs. A CoC value of 1 indicates a lack of interference, while values significantly <1 represent strong interference. The CO interference was measured at three consecutive intervals (*URA3-LEU2-LYS2, LEU2-LYS2-ADE2* and *LYS2-ADE2-HIS3)* on the chromosome XV. The *hop1-K65A,N67Q/hop1-K65A,N67Q* strain did not show a CO interference defect in these intervals compared with WT. The CoC values in WT were 0.699 at *URA3-LEU2-LYS2*, 0.805 at *LEU2-LYS2-ADE2* and 0.877 at *LYS2-ADE2-HIS3* compared to 0.809, 0.784 and 0.890, respectively, for the same genetic intervals in the *hop1-K65A,N67Q/hop1-K65A,N67Q* strain (Supplementary Table S8). Similar results were observed from the single spore data with CoC values of 0.728, 0.678 and 1.116 in WT compared to 0.807, 0.812 and 0.984 in the *hop1-K65A,N67Q/hop1-K65A,N67Q* strain (Supplementary Table S8). Alternatively, interference was measured by determining the ratio of observed number of nonparental ditype tetrads (NPDs) over expected number of NPDs. The interference values are expressed as the NPD ratio: an NPD ratio of <1 is indicative of positive CO interference. The NPD ratios for four intervals (*URA3-LEU2, LEU2-LYS2, LYS2-ADE2* and *ADE2-HIS3*) are shown in the *hop1-K65A,N67Q/hop1-K65A,N67Q* strain relative to WT, except the *ADE2-HIS3* interval (Supplementary Table S9).

### ChIP-seq analyses show reduced binding of the Hop1^K65A,N67Q^ variant to meiotic chromosomes

Given the *in vitro* DNA binding data (Figure 8), the obvious question was whether the ATPase activity modulates Hop1 binding to meiotic chromosomes. To investigate this possibility, calibrated ChIP-seq experiments were carried out to compare the genome-wide occupancy of WT and Hop1^K65A,N67Q^ variant using ChIP-grade anti-Hop1 antibodies as previously described (45,103). Before performing the ChIP-seq assays, western blots were performed on cell lysates derived from *HOP1/HOP1* and *hop1-K65A,N67Q/hop1-K65A,N67Q* strains at different times after induction of meiosis. We found comparable levels of WT Hop1 and Hop1^K65A,N67Q^ variant, which peaked at 4-h after meiosis induction, indicating that K65A and N67Q substitutions did not impact Hop1 expression during meiosis (Supplementary Figure S8). In the light of these results, we chose 4 h time point to perform ChIP-seq experiments.

**Figure 10.**
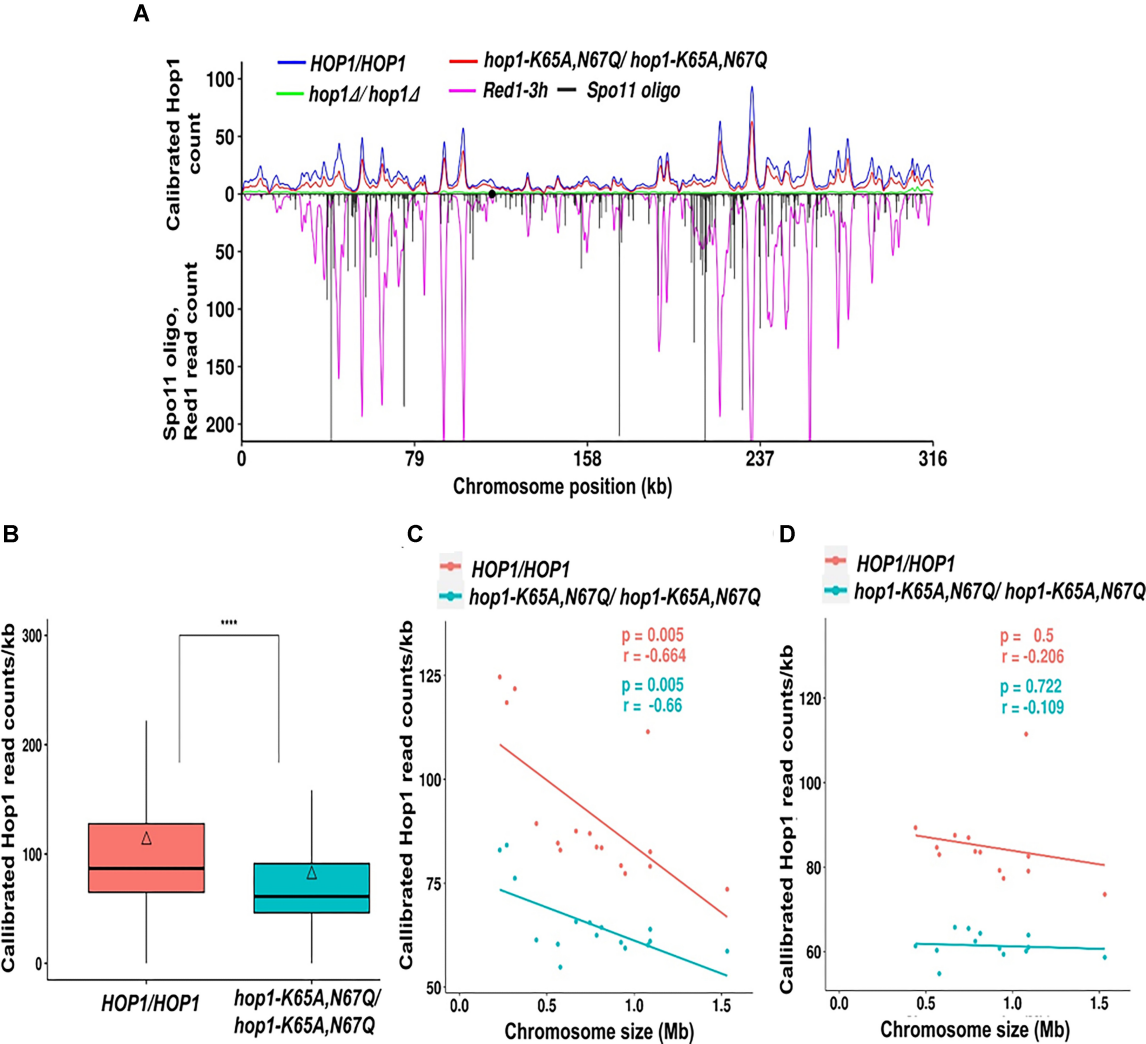
ChIP-seq data reveals reduced association of Hop1^K65A,N67Q^ variant across the genome. (**A**) Hop1 occupancy level in the WT, *hop1-K65A,N67Q/hop1-K65A,N67Q* and *hop1Δ/hop1Δ* strains at the 4-h time point on chromosome III. The Spo11 and Red1 binding data are from previous publications (26,136). Black circle represents the location of the centromere. (**B**) The box plot represents the genome-wide Hop1 read density in WT and the *hop1-K65A,N67Q/hop1-K65A,N67Q* strains. (**C**) Line graph shows the Hop1 read density (reads per million/kb) as a function of chromosome size. (**D**) Linear graph represents the exclusion of the short chromosomes (I, III and VI). The p values indicate statistical significance, and the Karl Pearson’s method calculates the correlation coefficient (*r*). The orange and light blue lines represent WT and the *hop1-K65A,N67Q/hop1-K65A,N67Q* mutant, respectively.

We performed ChIP-seq assays on WT and the *hop1-K65A,N67Q/hop1-K65A,N67Q* mutant cells, using *hop1Δ/hop1Δ* strain as internal control. Our results showed that ChIP peaks in the WT strain overlapped with *hop1-K65A,N67Q/hop1-K65A,N67Q* strain (Figure 10A and Supplementary Figure S9), which also correlate with the Red1 ChIP-seq peaks (25,26,45). Importantly, ChIP-seq analysis revealed markedly reduced binding of the Hop1^K65A,N67Q^ variant compared with the WT species (Figure 10A and Supplementary Figure S9); this inference was supported by statistical analysis (Figure 10B). The Hop1 read density was greater in the WT (mean: 112.92 reads/kb; median: 86.91 reads/kb) than the Hop1^K65A,N67Q^ variant (mean: 81.28 reads/kb; median: 61.24 reads/kb) (Figure 10B), which is statistically significant (****P< 0.0001, Wilcoxon rank sum test).

Previous studies have shown that shorter chromosomes exhibit higher DSB densities and CO frequencies than larger chromosomes (26,136–138). Moreover, it has been demonstrated that Hop1 exhibits preferential enrichment on shorter chromosomes. This, in turn, leads to increased density of meiotic DSBs on shorter chromosomes (25,26,139). Thus, we compared the read density of Hop1 across chromosomes in the *HOP1/HOP1* and *hop1-K65A,N67Q/hop1-K65A,N67Q* strains by regression analysis using a linear model. In this analysis, the correlation coefficients for *HOP1/HOP1* (*r* = –0.664) and *hop1-K65A,N67Q/hop1-K65A,N67Q* strain (*r* = –0.66) were negative and statistically significant (*P* = 0.005) (Figure 10C). The negative correlation coefficients indicate an inverse relationship between the Hop1 read density and chromosome size in both *HOP1/HOP1* and the *hop1-K65A,N67Q/hop1-K65A,N67Q* strains. These results suggest that the K65A and N67Q substitutions do not affect the increased enrichment of Hop1 on the three smallest chromosomes with unusually high DSB density. However the correlation coefficients for Hop1 read density (*r* = –0.206) in the *HOP1/HOP1* and *hop1-K65A,N67Q/hop1-K65A,N67Q* strains (*r* = –0.109) were not significant when the three smallest chromosomes (I, III and VI) were excluded (Figure 10D). Together, our data and analysis suggest that the enrichment of Hop1 on small chromosomes is specific to the chromosomes I, III and VI and is maintained in both *HOP1/HOP1* and the *hop1-K65A,N67Q/hop1-K65A,N67Q* strains.

We further sought to determine the binding of Hop1^K65A,N67Q^ variant at different sites on meiotic chromosomes. To achieve this, ChIP-quantitative PCR was performed in *HOP1/HOP1*, *hop1Δ/hop1Δ* and the *hop1-K65A,N67Q/hop1-K65A,N67Q* cells at two DSB hotspots (*BUD23* and *ECM3*), chromosome axes (*Axis I*, *Axis III*), centromere (*CEN VIII*) and a DSB cold spot (*YCR093W*) (102). The ChIP-qPCR data from two biological replicates showed reduced levels of binding of Hop1^K65A,N67Q^ variant to the hotspot *BUD23* and the centromere CEN VIII locus (Supplementary Figure S10). On the other hand, no enrichment of either WT Hop1 or Hop1^K65A,N67Q^ variant was seen at the chromosome axis (*Axis I*and *Axis III*), *ECM3* and DSB cold spot *YCR093W* sites.

## Discussion

In this study, we made an intriguing and unexpected discovery that Hop1 has a novel DNA-independent ATPase activity. This conclusion is in harmony with our finding that K65A and N67Q substitutions in the putative ATP-binding site synergistically abolished intrinsic ATPase activity of Hop1. Our data further reveal that the Hop1^K65A,N67Q^ variant displayed significantly reduced affinity for the HJ and meiotic chromosomes, while enhancing the frequency of COs, suggesting that this previously unrecognized function broaden the portfolio of mechanisms through which Hop1 may regulate various meiosis-specific processes. We discuss the implications of our findings below.

Although the components of the meiotic chromosome axis have been known since the 1980s, their biochemical and structural characterization remains largely understudied. We therefore set out to determine the biochemical activity of Hop1 and found that it interacts preferentially with the branched DNA structures, such as DNA flaps and the HJs over dsDNA and ssDNA, implying that its function entails a direct engagement with DNA recombination intermediates (61–63,66,70). In the current study, we unexpectedly discovered that Hop1 has DNA-independent ATPase activity. This inference was supported by the following experimental observations. Firstly, Hop1 pulled-down by anti-Hop1 antibody showed robust ATPase activity. Secondly, size-exclusion chromatography demonstrated co-elution of ATPase activity with Hop1 protein peak. Finally, Hop1 synthesized in a cell-free protein synthesis system exhibited robust ATP hydrolysis activity. The maximal ATPase activity of Hop1 was observed at pH 7.0, between 30 and 37°C, and in the presence of Mg^2+^/Ca^2+^. Further, kinetics analysis indicated that the Hop1’s ATPase activity displays a Hill coefficient of 1.3 ± 0.10 and an apparent *K*_m_ of ∼27 μM. Furthermore, various DNA substrates had no impact on the ATPase activity of Hop1, implying that this activity is DNA-independent. Curiously, Hop1 also catalyzed hydrolysis of GTP to the same extent as that of ATP, but how this contributes to its function remains unclear.

The question arises, whether the observed ATPase activity was due to a possible contaminant ATPase in the purified protein sample. However, the observation that K65A and N67Q substitutions in the putative ATP binding site completely abolished the ability of Hop1 to hydrolyze ATP, excluded the possibility that a contaminant ATPase was present in our preparation. Having said that, in the realm of structure activity studies, we cannot fully exclude at this stage the possibility that the reduced activity of Hop1^K65A,N67Q^ variant might be due to subtle conformational changes induced by substitution of residues K65 and N67 in the HORMA domain, although our results argue against such a possibility (Figure 5F and G). Curiously, our ChIP-seq data revealed a notable correlation between reduced affinity of Hop1^K65A,N67Q^ variant for the HJ and its association with meiotic chromosomes but reciprocally increased the CO frequency by ∼1.2-fold. While this demonstrates the biological significance of ATPase activity to the functions of Hop1, the molecular basis for small, but statistically significant increase in CO frequency is unclear, although it is conceivable that the regulation of CO frequency is multifactorial, serving to safeguard against inappropriate segregation of chromosomes during meiosis (4). Consistent with this notion, previous studies have shown that other genetic factors, including Zip1, Zip2, Zip3, Zip4, Spo16, Mer3, Msh4 and Msh5, of the ZMM-dependent CO pathway also regulate the frequency and distribution of meiotic COs across different loci in the genome (140–142). These findings provide a likely explanation for why we found a small increase in CO frequency in the *hop1-K65A,N67Q/hop1-K65A,N67Q* strain.

Probing further the mechanism by which Hop1 hydrolyses ATP, our integrative approach suggested that both N- and C-terminal regions of Hop1 contain residues that are likely to be involved ATP binding and/or hydrolysis. Intriguingly, we observed that mixing the N- and C-terminal fragments of Hop1 restored the ATPase activity to near WT levels. We validated our results, demonstrating the interaction *in trans* between the N- and C-terminal regions of Hop1 using MST and far-western blot analyses. These results imply that the active site may be created when Hop1 interacts with ATP and interdependence between the N- and C-terminal domains within the protein. This is in line with previous studies demonstrating that inter-domain interactions facilitate the ATPase activity of p97/VCP (143), DEAD-box proteins, DDX3 (144), Dhh1 (145) and *E. coli* Hsp70 (146). To complement our *in vitro* experiments, the CO frequencies were measured at four consecutive loci in the *hop1-K65A,N67Q/hop1-K65A,N67Q* strain and found a 1.2-fold increase in CO frequency in the mutant relative to WT. We posit that the enhanced CO frequency in the Hop1^K65A,N67Q^ variant activity might be due to the disruption in the organization of the meiotic chromosome axis. Such disruptions in chromosome axis may result in enhanced COs due to changes in the CO:non-CO ratio or an alteration in the DSB frequency. At present, we cannot distinguish between these possibilities. Regardless of the precise mechanism, our results suggest that the ATPase activity of Hop1 plays an important role in regulating CO frequency.

We note that a recent study (147) has found reduced number of COs in *asy1* mutants, the *Arabidopsis* homolog of *S. cerevisiae HOP1*. Likewise, studies have shown that the *S. cerevisiae hop1^SCD^* and *S. pombe hop1Δ* strains exhibit reduced levels of COs (29,46). On the other hand, our results are consistent with a previous study which found a ∼1.5-fold increase in CO levels in the *pch2Δ/pch2Δ* strain compared to the WT (143,135,148). It is also interesting to point out that, we observed no CO interference defects in the *hop1-K65A,N67Q/hop1-K65A,N67Q* strain, probably because the increase in the CO frequency was modest. Given these findings, further studies are needed to elucidate the molecular mechanism by which the loss of ATPase activity of Hop1 elevates CO frequency in *S. cerevisiae*. Looking forward, it will be interesting to explore whether the Hop1 ATP-deficient variant synergizes with other core components of SC to impact the CO frequency.

Nonetheless, it is also possible that the Hop1 ATPase activity may control its interaction with other meiosis-specific factors, such as Red1 (28,40,149), Mek1 (34,35,150) and Pch2 (23,32,148,151) or mitigate promiscuous non-functional interactions. Interestingly, previous studies have demonstrated that Hop1 is extensively modified by phosphorylation and SUMOylation, which have been shown to regulate Hop1 functions (28,29,152,153). Thus, the finding that Hop1 has an intrinsic DNA independent ATPase activity introduces an additional level of control to the regulation of its meiosis-specific functions.

It is interesting to note that there are notable similarities between Hop1 and Pch2 in their ATPase activities. Like Hop1, Pch2 possesses an inherent DNA-independent ATPase activity (23). Further, Hop1 and Pch2 play crucial roles in promoting the interhomolog bias, a process wherein DSBs are repaired preferentially using the homologous template instead of a sister chromatid (154). However, an apparent paradox is that Pch2 acts antagonistically to evict Hop1 from DNA in an ATP hydrolysis-dependent fashion (23,31), consistent with genetic studies that the Hop1 localization on meiotic chromosomes is negatively regulated by Pch2 (31,32,155). One possible explanation for these findings is that Pch2 might dislodge promiscuously loaded Hop1 from meiotic chromosomes while allowing Hop1 binding to specific DNA sequences and/or structures at interhomolog CO sites. Further, we speculate that Hop1 may use ATP hydrolysis as an energy source to regulate chromatin/nucleosome remodeling at or near the recombination sites, consistent with the finding that Hop1 directly interacts with nucleosomal DNA (48).

It was notable that the high-resolution ChIP-seq analysis revealed that both Hop1 WT and the Hop1^K65A,N67Q^ variant associate with similar sites on the meiotic chromosomes, including the smaller chromosomes I, III and VI (Figure 10). However, consistent with our *in vitro* DNA binding data, the extent of binding of the Hop1^K65A,N67Q^ variant to the genomic DNA sits was significantly reduced compared with the WT (Figure 10). Viewed together, our study provides novel insights into Hop1 function and supports a model wherein reduced chromosomal association of the Hop1^K65A,N67Q^ variant may contribute to an increase in the CO frequency. Molecular details and mechanisms that underlie these effects remain to be defined.

In summary, the main surprise was the finding that Hop1 possesses DNA-independent ATPase activity and the involvement of this activity in the regulation of meiotic COs. This study paves the way for future research on how the ATPase activity impacts the role of Hop1 in meiotic prophase checkpoint activation, formation of programmed DSBs and interaction with different meiosis-specific effector proteins. In line with this, investigations into the presumptive ATPase activity of Hop1 orthologs may provide further insights into the conservation of its regulatory role across different species.

## Supplementary Material

gkae1264_Supplemental_File

## Data Availability

ChIP-Seq data: NCBI Sequence Read Archive PRJNA1107023 (https://www.ncbi.nlm.nih.gov/sra/?term=PRJNA1107023).
