# Supplementary material for: The ATPase activity of yeast chromosome axis protein Hop1 affects the frequency of meiotic crossovers
Source: Nucleic Acids Res. 2024 Dec 27;53(3):gkae1264. doi: 10.1093/nar/gkae1264 (PMC11797056; doi:10.1093/nar/gkae1264)
Supplement: gkae1264_Supplemental_File [file gkae1264_supplemental_file.pdf]

Supplementary data

The ATPase activity of yeast chromosome axis protein Hop1 affects the frequency of meiotic crossovers

Supplementary Tables

Table S1. Primer sequences used for the generation of Hop1 mutants.

Table S2. DNA substrates used in the study.

Table S3. Oligonucleotides used for the prepartion of DNA substrates.

Table S4. Primers used for the generation of yeast strains.

Table S5. *S. cerevisiae* strains used in this study.

Table S6. Genetic map distances from tetrad and spore data in *HOP1/HOP1* and *hop1-K65A,N67Q/hop1-K65A,N67Q* strains, in the EAY1108/EAY1112 strain background.

Table S7. The percentage of aberrant segregation events observed in tetrads from *HOP1/HOP1* and *hop1-K65A,N67Q/h op1-K65A,N67Q* strains in EAY1108/EAY1112 strain background.

Table S8. Analysis of crossover interference in the *HOP1/HOP1* and *hop1-K65A,N67Q/hop1-K65A,N67Q* strains using CoC values.

Table S9. Analysis of CO interference in the *HOP1/HOP1* and *hop1-K65A,N67Q/hop1-K65A,N67Q* strains using NPD ratios.

Table S10. Primer sequences used for ChIP-qPCR.

Supplementary Figures

Figure S1. ATPase activity is associated with Hop1.

Figure S2. Determination of optimal assay conditions for Hop1 ATPase activity.

Figure S3. Thermal denaturation profiles of Hop1 in the presence of the indicated nucleotide moieties.

Figure S4. Hop1 lacks recognizable sequence motifs essential for ATP-binding and/or hydrolysis.

Figure S5. Bioinformatics analysis reveals possible interaction between Hop1 and ATP.

Figure S6. The Hop1 putative ATP-binding site is fairly conserved among distantly related species.

Figure S7. Hop1 HORMA interacts with Hop1CTD.

Figure S8. Western blot analysis of cell lysates at different time points after induction of meiosis.

Figure S9. Hop1 occupancy in the *S. cerevisiae* chromosomes of WT, *hop1-K65A,N67Q/hop1-K65A,N67Q* and *hop1Δ/hop1Δ* strains at the 4 h after induction of meiosis.

Figure S10. ChIP-qPCR analysis of Hop1 bound to the meiotic chromosomes at the indicated genomic sites.

Supplementary Tables

Table S1. Primer sequences used for the generation of Hop1 mutants.

Primer		Primer sequence (5'-3')
Hop1^{K65A}	Forward	CTACAATGCGCAAAACACG
	Reverse	CGTGTTTTGCGCATTGTAG
Hop1^{N67Q}	Forward	ATAAGCAACAAACGTCTC
	Reverse	GAGACGTTTGTTGCTTAT
Hop1^{K65A,N67Q}	Forward	AACTACAATGCGCAACAAACGTCTC
	Reverse	GAGACGTTTGTTGCGCATTGTAGTT
Hop1^{N139Q}	Forward	TAATGTCCAAATTAATGT
	Reverse	ACATTAATTTGGACATTA
Hop1^{R352A}	Forward	AAAGTTGCGCTAAAACAC
	Reverse	GTGTTTTAGCGCAACTTT
Hop1^{R558A}	Forward	GCCAAAAAAGGGCACGTTACG
	Reverse	CGTAACGTGCCCTTTTTTGGC

Table S2. DNA substrates used in the study.

DNA substrate	Oligonucleotides used	Structure
Holliday junction	HJ01, HJ02*, HJ03, HJ04	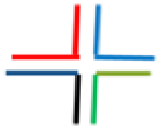
dsDNA	ODN1*, ODN2	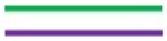
ssDNA	ODN2*	

*Asterisk indicates the oligonucleotides which were radiolabeled at their 5'-end.

Table S3. Oligonucleotides used for the preparation of DNA substrates.

Name of the oligonucleotide	Sequence (5'—3')	Length (nt)
HJ01	GACGCTGCCGAATTCTACCAGTGCCTTGCTAGGAC<br>ATCTTTGCCCACCTGCCGGTTCACCC	61
HJ02	TGGGTGAACCTGCAGGTGGGCAAAGATGTCCTAGC<br>AATGTAATCGTCAAAGCTTTATGCCGTT	62
[6FAM]HJ02	[6FAM]TGGGTGAACCTGCAGGTGGGCAAAGATGTCCTAG<br>C<br>AATGTAATCGTCAAAGCTTTATGCCGTT	62
HJ03	CAACGGCATAAAGCTTGACGATTACATTGCTAGGAC<br>ATGCTGTCTAGAGGATCCGACTATCGA	63
HJ04	ATCGATAGTCGGATCCTCTAGACAGCATGTCCTAGC<br>AAGGCACTGGTAGAATTCGGCAGCGT	62

ODN1	GCCGTGATCACCAATGCAGATTGACGAACCTTTGCC CACGT	41
ODN2	ACGTGGGCAAAGGTTCGTCAATCTGCATTGGTGATCACG GC	41

Table S4. Primers used for the generation of yeast strains.

Primer		Primer sequence (5'-3')
FPDelHop1	Forward	ACACTTATAAACGGTTTAAAAACAGCTTTATCTCAGA AAAGTCAGGAATTATGCGTACGCTGCAGGTCGAC
RPDelHop1	Reverse	CTGTTGAAGAAGTGCCTGTTTAAATGATGAGCATAA TATAATTCGGTTTTCGCTAATCGATGAATTCGAGCTCG
Locus FP	Forward	ATGATTATTTCCATTGAAAATC
FPHop1 cassette	Forward	ACACTTATAAACGGTTTAAAAACAGCTTTATCTCAGAA AAGTCAGGAATTATGTCTAATAAACAACTAG
RPHop1-KanMX4	Reverse	CGACCTGCAGCGTACGCTACCAGTTACTTTTCAA
FPKanMX4	Forward	AAGTAACTGGTAGCGTACGCTGCAGGTCGAC

Table S5. *S. cerevisiae* strains used in this study.

Strain	Source	Genotype
KTY81/ EAY1108	Argueso *et al*., 2004	*MATa ho hisG, lys2, ura3, leu2 hisG,trp1 hisG, URA3-cenXVi, LEU2-chXVi, LYS2-chXVi*
KTY82/ EAY1112	Argueso *et al*., 2004	*MATα ho hisG,lys2, ura3, leu2 hisG, trp1 hisG, ade2 hisG, his3 hisG, TRP1-cenXVi*
KMY 54	This study	Derived by mating KTY81 and KTY82
KMY 63	This study	Derived from KTY81 *hop1 :: hphNT1*
KMY 56	This study	Derived from KTY82 *hop1 :: hphNT1*
KMY 64	This study	Derived by mating KMY 63 and KMY 56
KMY 65	This study	Derived from KTY81 *hop1-K65A,N67Q:: kanMX4*
KMY 66	This study	Derived from KTY82 *hop1-K65A,N67Q:: kanMX4*
KMY 68	This study	Derived by mating KMY65 and KMY66
NHY1162	Martini *et al*., 2006	*MATα ho::hisG,leu2::hisG,ura3(Δsma-pst),his4-X::LEU2-(NgoM IV-URA3)*
NHY1168	Martini *et al*., 2006	*MATa, ho::hisG, leu::hisG, ura3(ΔSma-Pst), HIS4::LEU2-(BamHI)*
KMY69	This study	Derived from NHY1162 *hop1::hphNT1*
KMY70	This study	Derived from NHY1168 *hop1::hphNT1*
KMY71	This study	Derived by mating KMY69 and KMY70
KMY72	This study	Derived from NHY1162 *hop1-K65A,N67Q:: kanMX4*

KMY73	This study	Derived from NHY1168 *hop1-K65A,N67Q:: kanMX4*
KMY74	This study	Derived by mating KMY72 and KMY73

Table S6. Genetic map distances from tetrad and spore data in *HOP1/HOP1* and *hop1-K65A,N67Q/hop1-K65A,N67Q* strains, in the EAY1108/EAY1112 strain background.

A. Tetrad data

Genotype	N	PD	TT	NPD	cM	S.E.	Fold increase	p values
URA3-LEU2								
HOP1/HOP1	344	210	121	3	20.8	1.9	-	-
hop1-K65A,N67Q/ hop1-K65A,N67Q	323	173	143	2	24.4	1.8	1.17	0.005
LEU2-LYS2								
HOP1/HOP1	344	149	182	3	29.9	1.9	-	-
hop1-K65A,N67Q/ hop1-K65A,N67Q	323	120	194	4	34.3	2.1	1.14	0.042
LYS2-ADE2								
HOP1/HOP1	344	242	90	2	15.3	1.7	-	-
hop1-K65A,N67Q/ hop1-K65A,N67Q	323	232	82	4	16.7	2.1	1.09	0.383
ADE2-HIS3								
HOP1/HOP1	344	123	200	11	39.8	2.9	-	-
hop1-K65A,N67Q/ hop1-K65A,N67Q	323	73	225	20	54.2	3.7	1.36	<0.0001

Standard error (S.E.) around the genetic distances (cM) was calculated using the Stahl Laboratory Online Tools website https://elizabethhousworth.com/StahlLabOnlineTools. N: four viable spore tetrads analysed. PD, Parental ditypes; TT, Tetratypes; NPD, Non-parental ditypes. Fold increase was calculated w.r.t. *HOP1/HOP1* strain. p-values were calculated using two-tailed G-test (degree of freedom = 2) (https://www.biostathandbook.com/gtestgof.html).

B. Spore data

Genotype	n	Par.	Rec.	cM	95% C.I.	Fold increase	p values
URA3-LEU2							
HOP1/HOP1	1563	1260	303	19.4	17.5-21.4	-	-
hop1-K65A,N67Q/ hop1-K65A,N67Q	1600	1217	383	23.9	21.9-26.0	1.23	<0.0001
LEU2-LYS2							
HOP1/HOP1	1563	1131	432	27.6	25.4-29.9	-	-
hop1-K65A,N67Q/ hop1-K65A,N67Q	1600	1103	497	31.1	28.8-33.3	1.12	0.002
LYS2-ADE2							
HOP1/HOP1	1563	1323	240	15.4	13.6-17.2	-	-
hop1-K65A,N67Q/ hop1-K65A,N67Q	1600	1362	238	14.9	13.2-16.7	N/A	0.593
ADE2-HIS3							
HOP1/HOP1	1563	1038	525	33.6	31.2-35.9	-	-

hop1-K65A,N67Q/ hop1-K65A,N67Q	1600	937	663	41.4	39.0-43.8	1.23	<0.0001

Genetic map distances (cM) were calculated by multiplying recombination frequencies (number of recombinant spores/total number of spores) by 100. 95% confidence interval (C.I.) for genetic map distance were determined using VassarStats (http://vassarstats.net/) n: number of single spores, Par., parental single spores; Rec., recombinant single spores. p-values were calculated using two-tailed G-test (https://www.biostathandbook.com/gtestgof.html).

Table S7. The percentage of aberrant segregation events observed in tetrads from *HOP1/HOP1* and *hop1-K65A,N67Q/hop1-K65A,N67Q* strains in EAY1108/EAY1112 strain background.

Genotype	Tetrads	All markers	*TRP1*	*URA3*	*LEU2*	*LYS2*	*ADE2*	*HIS3*
HOP1/HOP1	344	2.9	0	0	0	0.6	2	0.3
hop1-K65A,N67Q/hop1-K65A,N67Q	323	1.8	0	0	0	0	0.9	0.9

Table S8. Analysis of crossover interference in the *HOP1/HOP1* and *hop1-K65A,N67Q/hop1-K65A,N67Q* strains using CoC values.

Genotype	Coefficient of coincidence (CoC)					
	URA3-LEU2-LYS2		*LEU2-LYS2-ADE2*		*LYS2-ADE2-HIS3*	
	Tetrads	Spores	Tetrads	Spores	Tetrads	Spores
HOP1/HOP1	0.699 (48/68.7)	0.728 (61/83.7)	0.805 (41/51)	0.678 (45/66.3)	0.877 (51/58.1)	1.116 (90/80.6)
hop1-K65A,N67Q/hop1-K65A,N67Q	0.809 (73/90.3)	0.807 (96/119)	0.784 (42/53.5)	0.812 (60/73.9)	0.890 (59/66.3)	0.984 (97/98.6)

CoC = Observed double crossovers/Expected double crossovers.

Table S9. Analysis of CO interference in the *HOP1/HOP1* and *hop1-K65A,N67Q/hop1-K65A,N67Q* strains using NPD ratios.

Genotype	Non-parental ditype (NPD) ratio			
	URA3-LEU2	*LEU2-LYS2*	*LYS2-ADE2*	*ADE2-HIS3*
HOP1/HOP1	0.428 (3/7)	0.136 (3/22)	0.667 (2/3)	0.367 (11/30)
hop1-K65A,N67Q/hop1-K65A,N67Q	0.167 (2/12)	0.129 (4/31)	1.24 (4/3)	N/A

NPD ratio = NPD observed/NPD expected. N/A indicates frequency of Tetratypes approaches the 2/3 limit and therefore the estimate is not reliable.

Table S10. Primer sequences used for ChIP-qPCR

Oligo name	Oligo sequence	Chromosome
AXIS I F	5’ AGTTTCGCCCACTGCCCAATA 3'	XV
AXIS I R	5' AGGTTCGTTATTGCGTTTGCC 3'	XV
AXIS III F	5' AGATCGGCAACCCATTACCC 3'	III
AXIS III R	5' GTGCTCCTGACGCAACATTC 3'	III
BUD23 F	5' GCTGTGCAATGCGGACACTTC 3'	III
BUD23 R	5' TATGTCGTCCACCTGGTCGTC 3'	III
ECM3 F	5' GACAATGACACACATCACAC 3'	XV
ECM3 R	5' CGGTTAAGATGTTCATCTTGC 3'	XV
CEN VIII F	5' ACGATCGGGATTAGCTGGAG 3'	VIII
CEN VIII R	5' AGTGAGCCTGCGTACTTTGT 3'	VIII
YCR093W F	5' GGTGGCAACCAAAGTCAAACC 3'	III
YCR093W R	5' CGGTATGTGGCCGATAGCATG 3'	III

Supplementary Figures

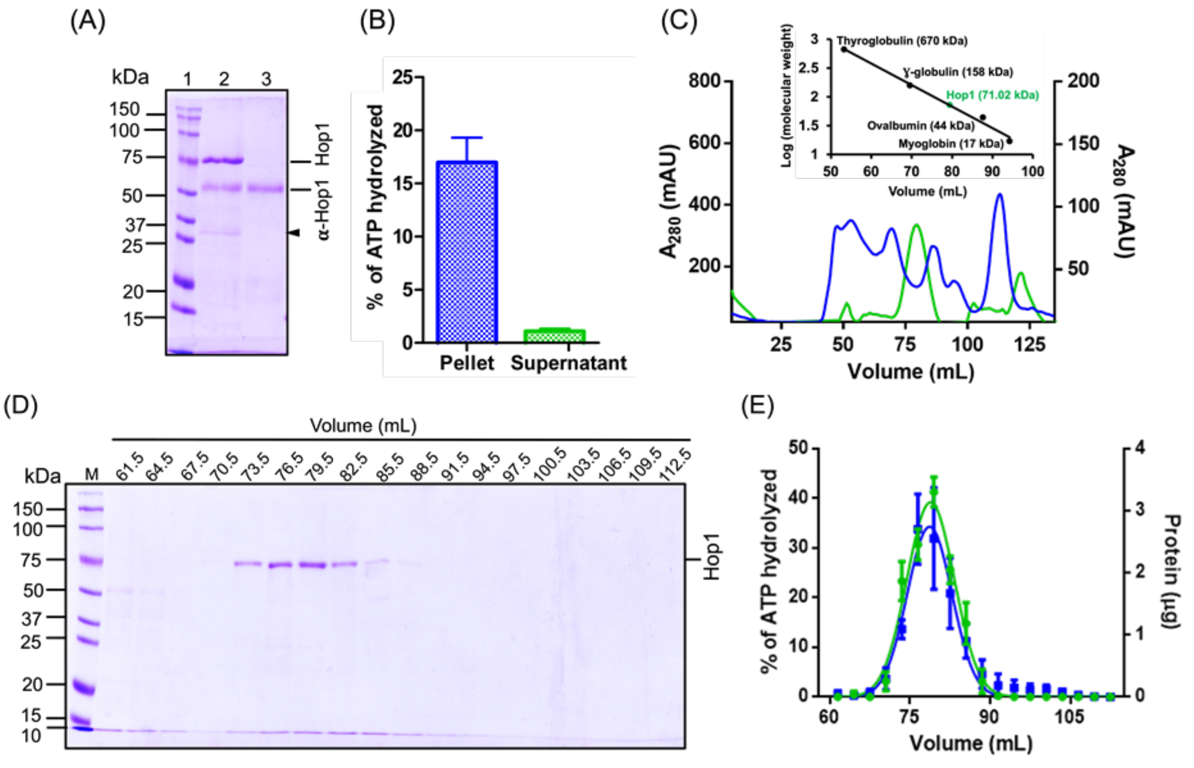

Figure S1. ATPase activity is associated with Hop1. (**A**) Immunoprecipitation of Hop1 with an anti-Hop1 antibody. Immunoprecipitated Hop1 was analysed by SDS-PAGE and staining with Coomassie brilliant blue. Lane 1, molecular weight standards; lane 2, pellet fraction; lane 3, anti-Hop1 antibodies (2.5 µg). Closed arrowhead indicates Hop1 degradation product. (**B**) Hop1 immunoprecipitated with anti-Hop1 antibody catalyzes ATP hydrolysis. The reaction mixtures containing supernatant and pellet fractions were incubated with 100 µM ATP. ATPase activity was monitored using malachite green colorimetric assay (n = 3). (**C**) UV absorbance values at 280 nm for standard molecular weight markers (blue trace) and Hop1 (green trace) during gel-filteration chromatography. Standard curve used to obtain the molecular weight of Hop1 is provided as an inset to the figure. (**D**) SDS-PAGE analysis of column fractions. (**E**) Co-elution of Hop1 protein and ATPase activity during gel-filtration chromatography. Purified Hop1 protein (25 µg) was applied to a Superdex 200 column and fractions of 1.5 ml were eluted. The indicated fractions were assayed for ATPase activity. The total protein was estimated by the Bradford assay. Green and blue traces correspond to Hop1 protein peak and ATPase activity, respectively. The error bars represent standard deviation (n=3).

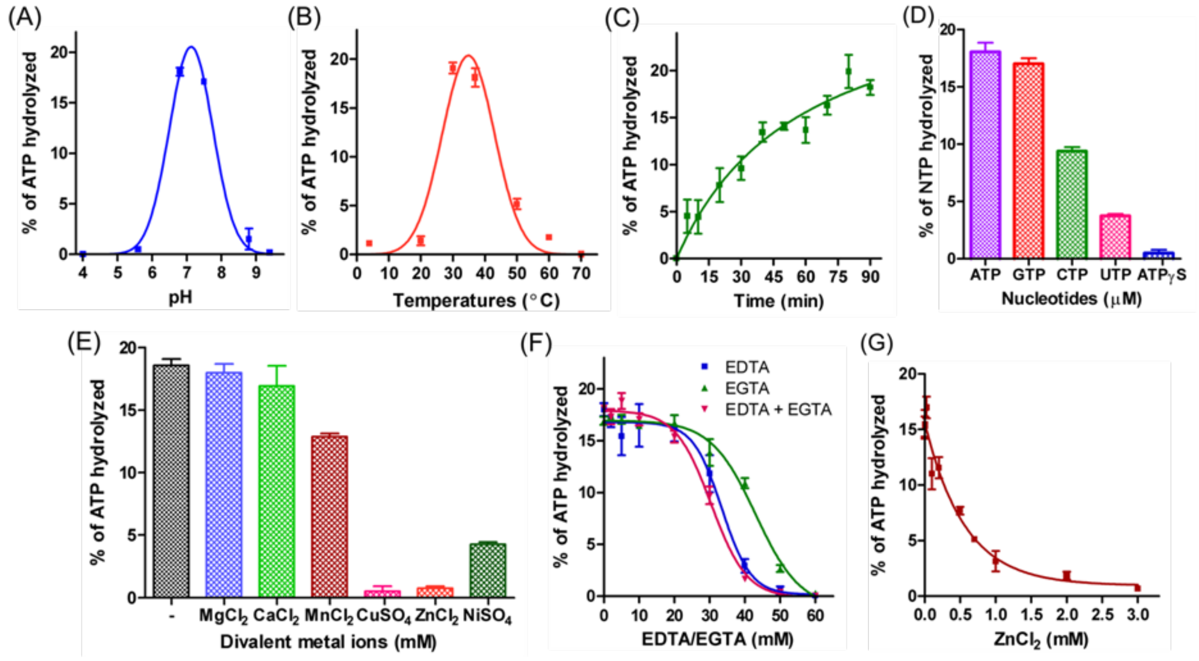

Figure S2. Determination of optimal assay conditions for Hop1 ATPase activity. ATPase activity at (**A**) different pH values, (**B**) different temperatures, (**C**) different periods of incubation, (**D**) ATPase activity with equivalent concentrations of different NTPs (100 µM) and **(E)** with different divalent cations (5 mM). (**F**) and (**G**) correspond to Hop1 ATPase activity as a function of equivalent concentrations of EDTA/EGTA, and at indicated concentrations of $ZnCl_2$, respectively. ATP hydrolysis was measured following co-incubation of Hop1 (200 nM) and ATP (100 µM) using a malachite green dye-binding assay. Error bars represent standard deviation (n=3).

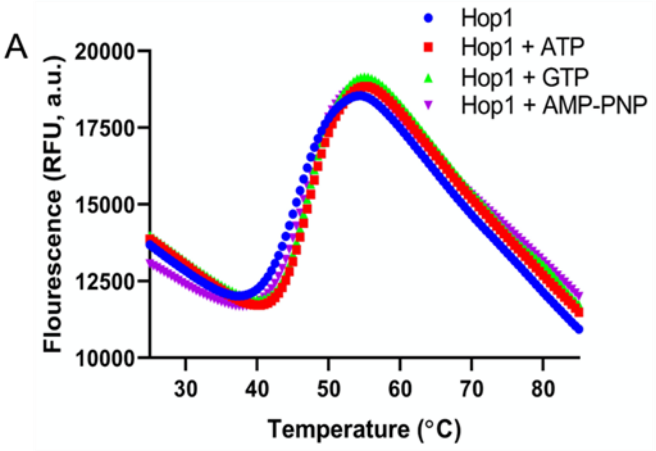

B

Protein	Melting temperature (T_m, °C)
Hop1	45.5 ± 0.5
Hop1 + ATP	47.5 ± 0.5
Hop1 + GTP	47 ± 0.5
Hop1 + AMP-PNP	46.5 ± 0.5

Figure S3. Thermal denaturation profiles of Hop1 in the presence of the indicated nucleotide moieties. (**A**) Thermal unfolding of Hop1 in the absence or presence of different nucleotides. Ten µg of Hop1 was mixed with 0.5X SYPRO Orange, and incubated with or without 5 mM of ATP, GTP or AMP-PNP. The raw fluorescence data was measured from 25 to 90 °C. (**B**) Table shows T_m values for each curve, determined as described in the Methods. For WT Hop1, the data from Figure 7F is used here for comparison.

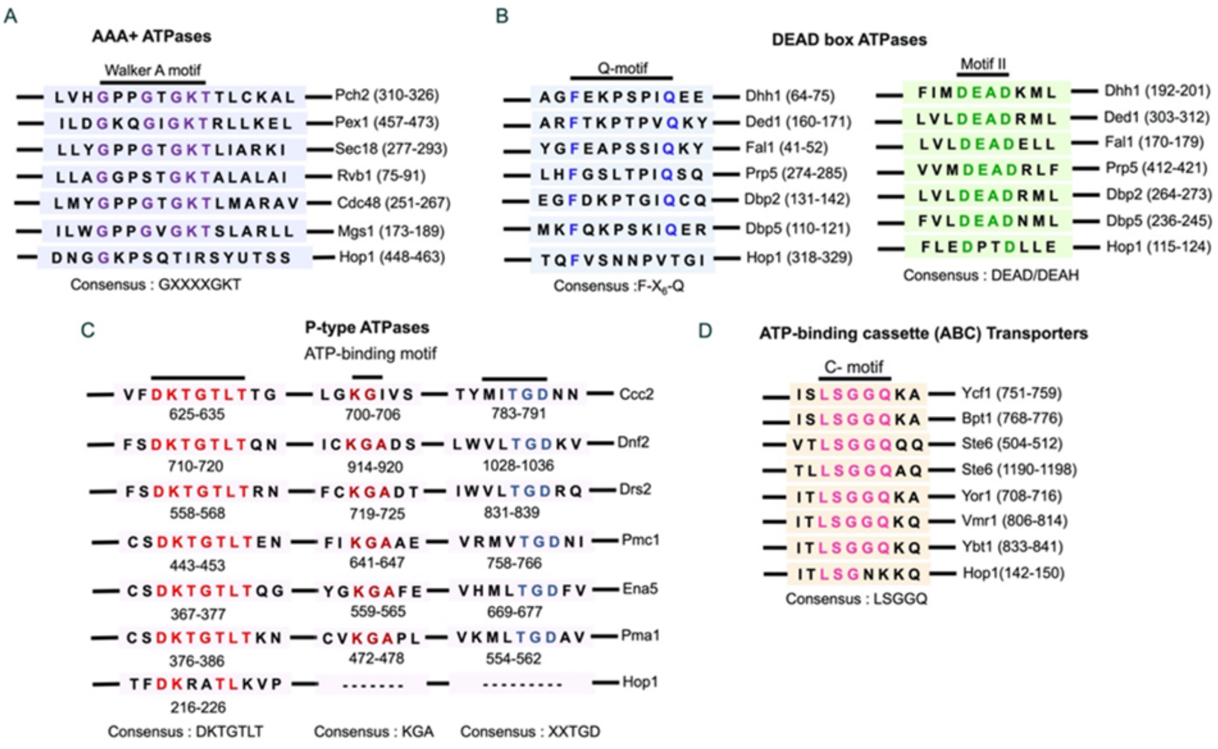

Figure S4. Hop1 lacks recognizable sequence motifs essential for ATP-binding and/or hydrolysis. Panels **A-D** show schematic representation of the consensus sequences corresponding to the Walker A motif, DEAD box, P-type ATPases or ABC transporters, respectively, in the specified proteins. The motifs were aligned using Clustal omega (https://www.ebi.ac.uk/jdispatcher/msa/clustalo). Fully conserved residues are highlighted and colored.

A. Top hits from the Cofactor and Coach ligand prediction servers

Top Hits	C-score	PDB Hit	Ligand family
1	0.07	3cmvA	ANP
2	0.06	3cmvF	ANP

B. Gene ontology server prediction

GO annotation	C-score	Family
GO:0032559	0.48	adenyl ribonucleotide binding
GO:0016462	0.47	purine ribonucleoside triphosphatase
GO:0035639	0.47	pyrophosphatase

Figure S5. Bioinformatics analysis reveals possible interaction between Hop1 and ATP. (**A**) Functional annotations of Hop1 by COFACTOR and COACH servers, based on I-Tasser structure prediction. Two top-ranked hits are shown (3cmv: Structure of *Escherichia coli* RecA-ssDNA and RecA-heteroduplex filaments) wherein C-score represents the confidence score for the prediction. Details of the members of ANP family of molecules was curated from BioLip database (Yang et al, 2013). (**B**) Gene ontology-based predictions for Hop1 function.

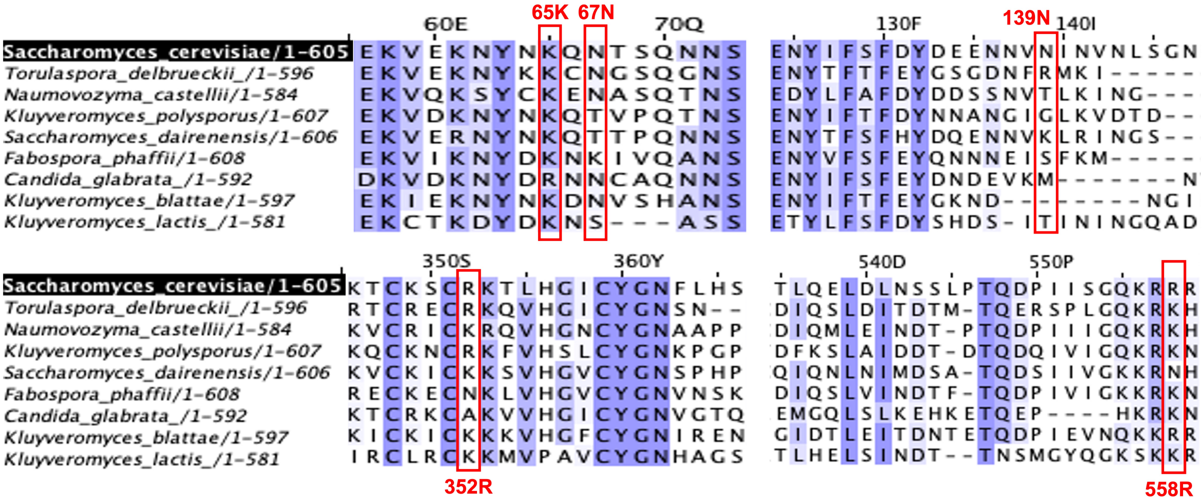

Figure S6. The Hop1 putative ATP-binding site is fairly conserved among distantly related species. Primary sequence of *S. cerevisiae* Hop1 protein (Accession: QHB09305) was submitted to the Jpred protein secondary structure prediction server (https://www.compbio.dundee.ac.uk/jpred/). The alignment was displayed using Jalview software (version 2.11.2.5). Residues in *Sc*Hop1 ATP-binding pocket (K65, N67, N139, R352 and R558) are marked in red boxes and colored based on percentage identity.

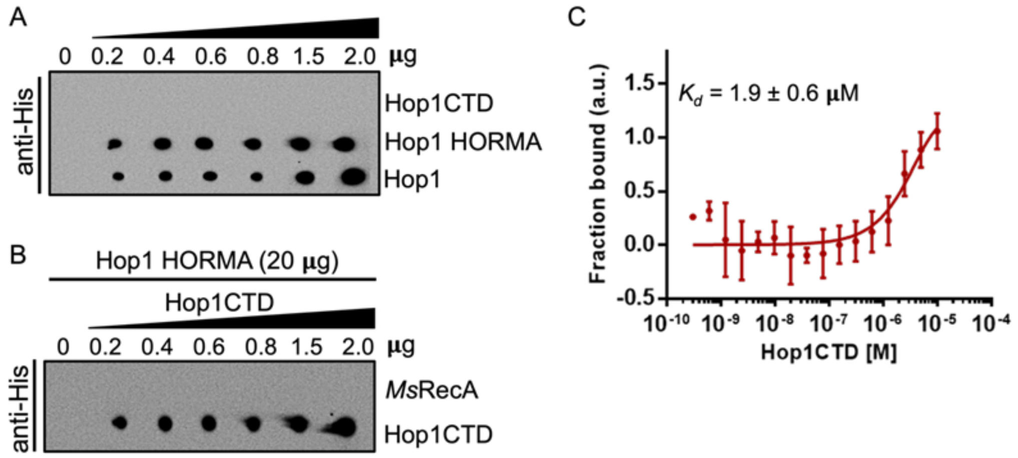

Figure S7. Hop1 HORMA interacts with Hop1CTD. (**A**) Increasing concentrations of either Hop1, Hop1CTD or Hop1 HORMA were spotted onto nitrocellulose membranes and probed using anti-His antibodies (upper panel). (**B**) Far-western analysis of Hop1 CTD-HORMA interactions. Increasing concentrations of Hop1CTD or *Mycobacterium smegmatis* RecA were spotted onto the membrane, followed by incubation with Hop1 HORMA (lower panel). (**C**) MST binding isotherm obtained upon incubating different concentrations of Hop1CTD with Red-tris-NTA labelled Hop1 HORMA. Data is representative of three independent experiments.

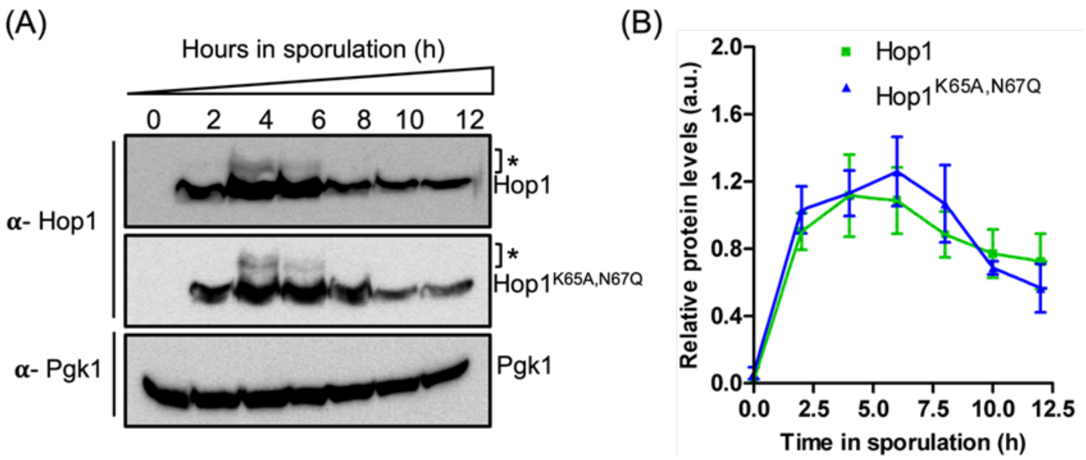

Figure S8. Western blot analysis of cell lysates at different time points after induction of meiosis. (**A**) Equal amounts of protein extracts from cells expressing either WT Hop1 or Hop1^{K65A,N67Q} mutant were subjected to Western blot analysis using antibody against Hop1. PGK1 was monitored in parallel as a loading control using anti-PGK1 antibody. The post-translationally modified forms of WT Hop1 and Hop1^{K65A,N67Q} variant are marked with an asterisk. (**B**) Quantification of band intensities corresponding to WT Hop1 and Hop1^{K65A,N67Q} variant, with respect to PGK1, respectively. The bands were quantified using ImageJ (Version 1.53) and graphs were obtained using GraphPad Prism (Ver 4.0). y-axis represents relative protein levels in arbitrary units and x-axis represents hours in the sporulation media. Data was obtained from three independent experiments.

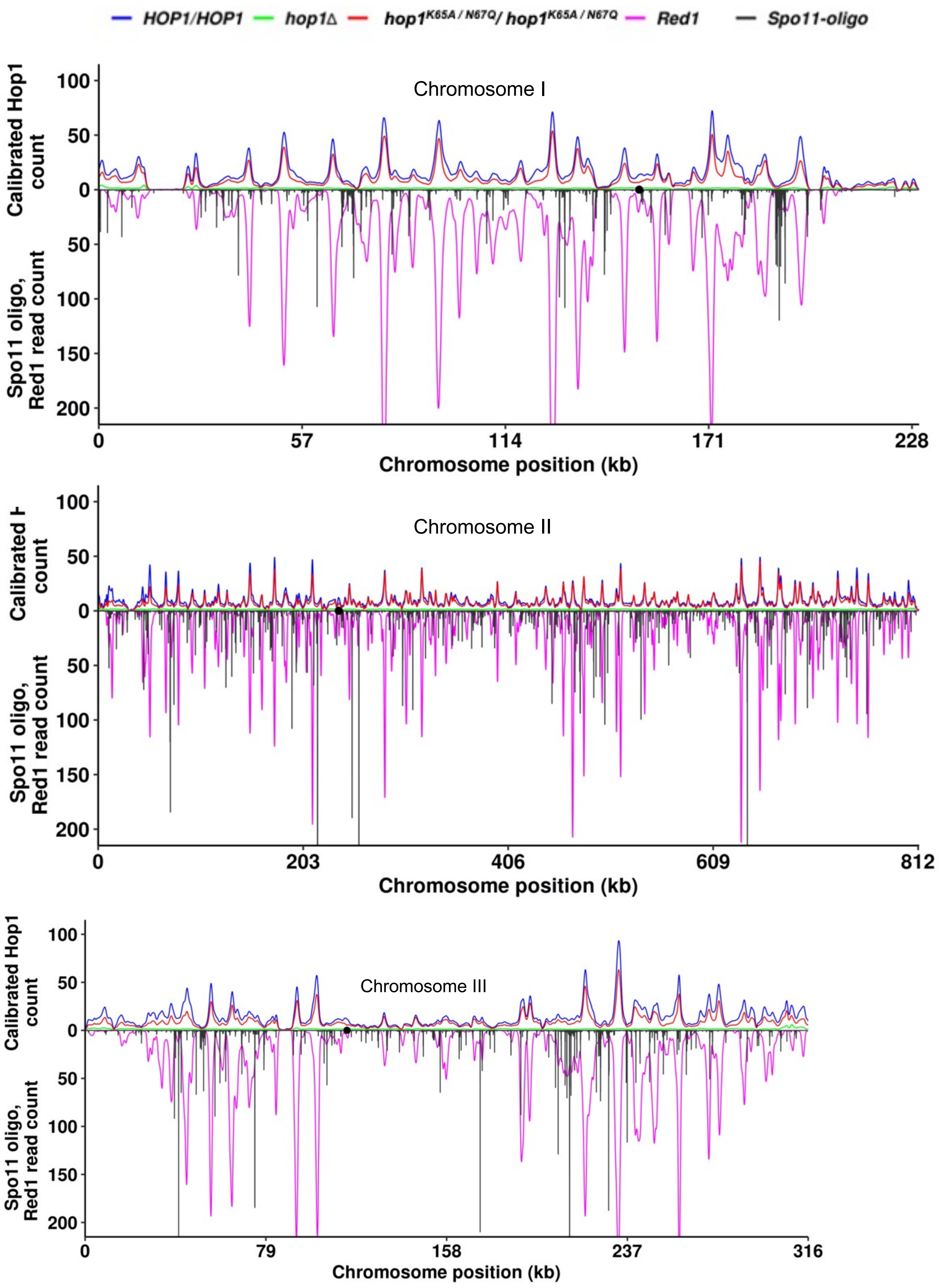
HOP1/HOP1
hop1Δ
hop1K65A / N67Q/ hop1K65A / N67Q
Red1
Spo11-oligo
Chromosome I
Calibrated Hop1 count
Spo11 oligo, Red1 read count
Chromosome position (kb)
Chromosome II
Chromosome III

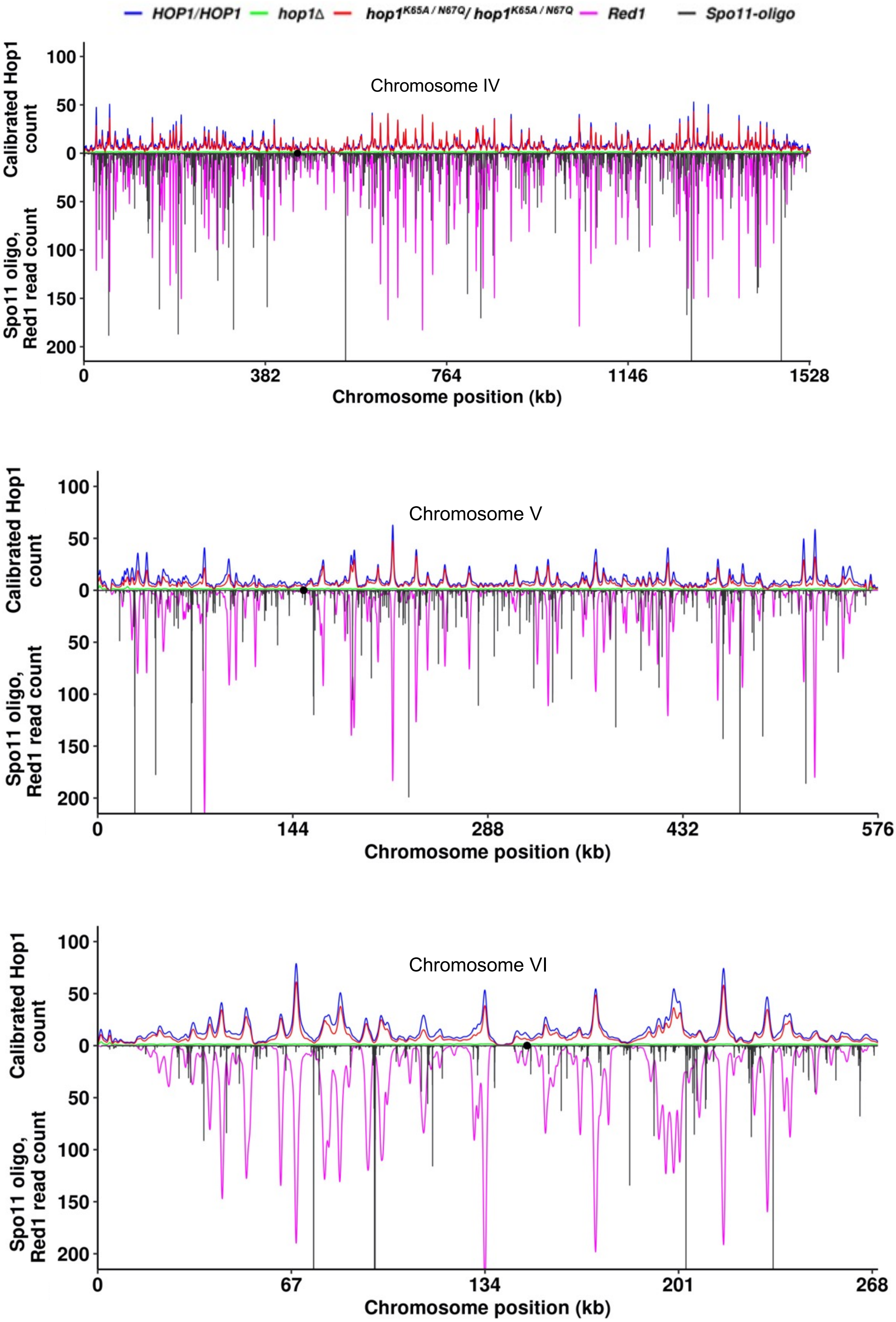
HOP1/HOP1
hop1Δ
hop1K65A / N67Q/ hop1K65A / N67Q
Red1
Spo11-oligo
Chromosome IV
Calibrated Hop1 count
Spo11 oligo, Red1 read count
Chromosome position (kb)
Chromosome V
Chromosome VI

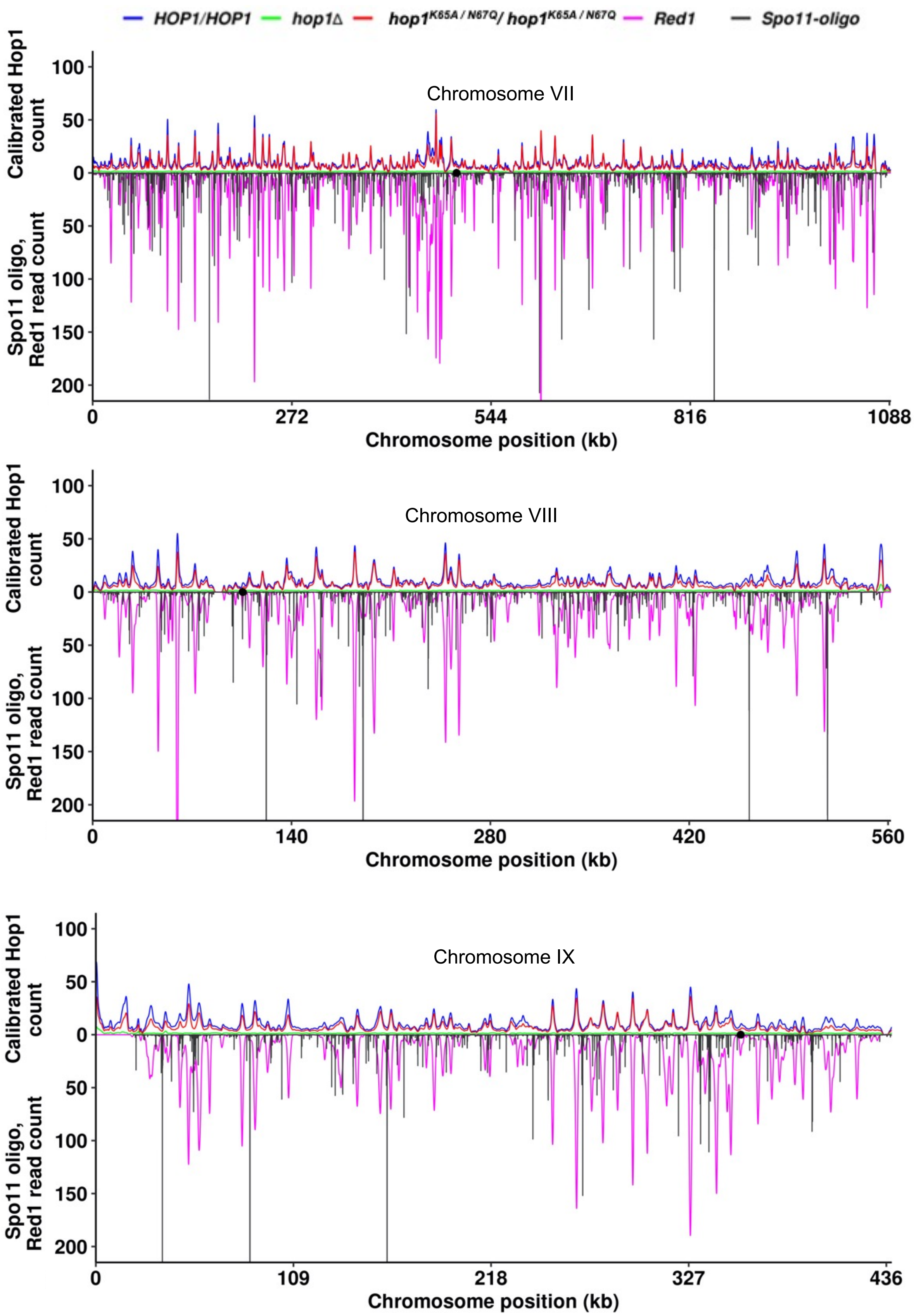
HOP1/HOP1
hop1Δ
hop1K65A / N67Q/ hop1K65A / N67Q
Red1
Spo11-oligo
Calibrated Hop1 count
Spo11 oligo, Red1 read count
Chromosome VII
0
50
100
150
200
0
272
544
816
1088
Chromosome position (kb)
Chromosome VIII
0
140
280
420
560
Chromosome position (kb)
Chromosome IX
0
109
218
327
436
Chromosome position (kb)

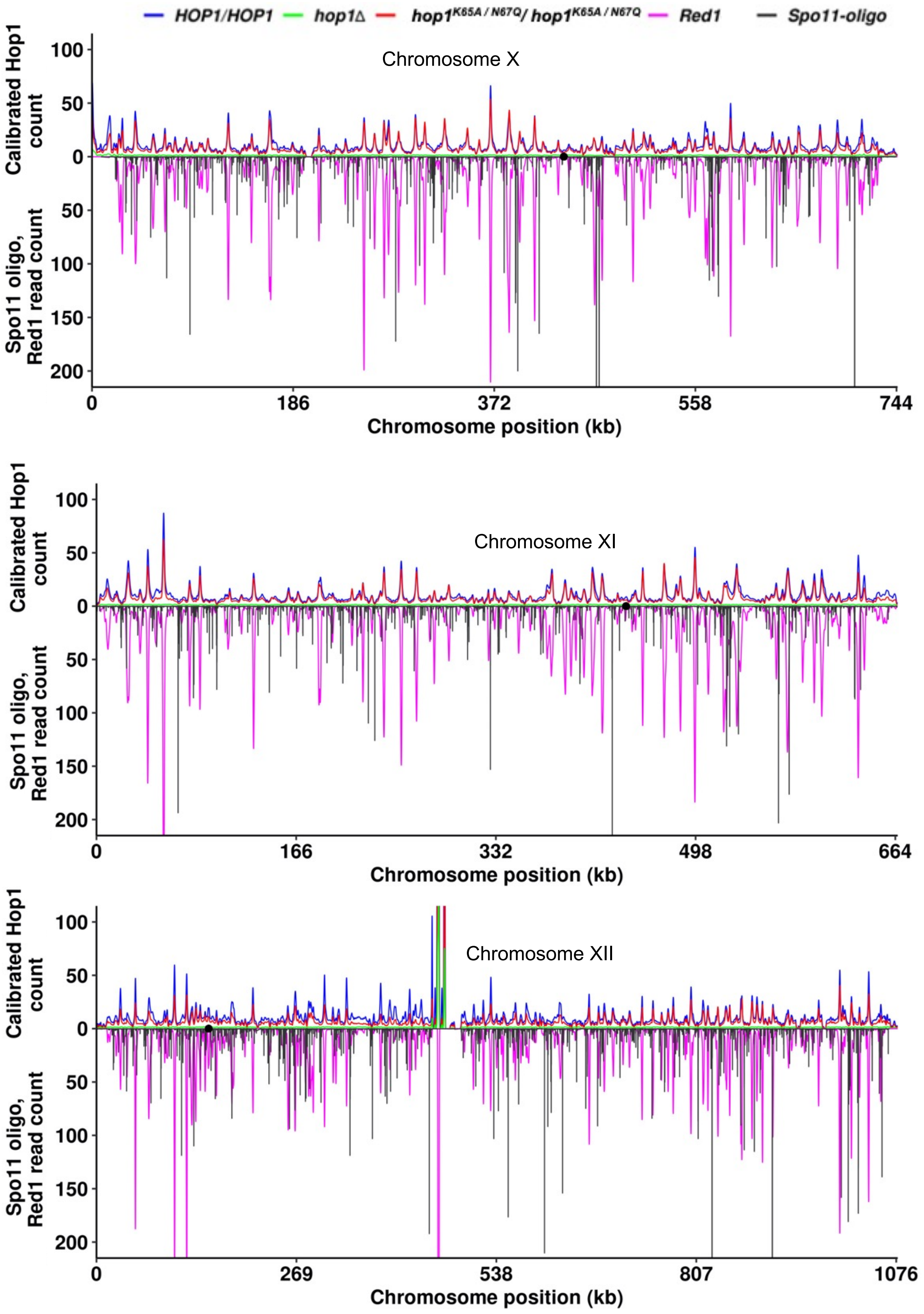
HOP1/HOP1
hop1Δ
hop1K65A / N67Q/ hop1K65A / N67Q
Red1
Spo11-oligo
Chromosome X
Chromosome XI
Chromosome XII
Calibrated Hop1 count
Spo11 oligo, Red1 read count
Chromosome position (kb)
0
186
372
558
744
166
332
498
664
269
538
807
1076
100
50
150
200

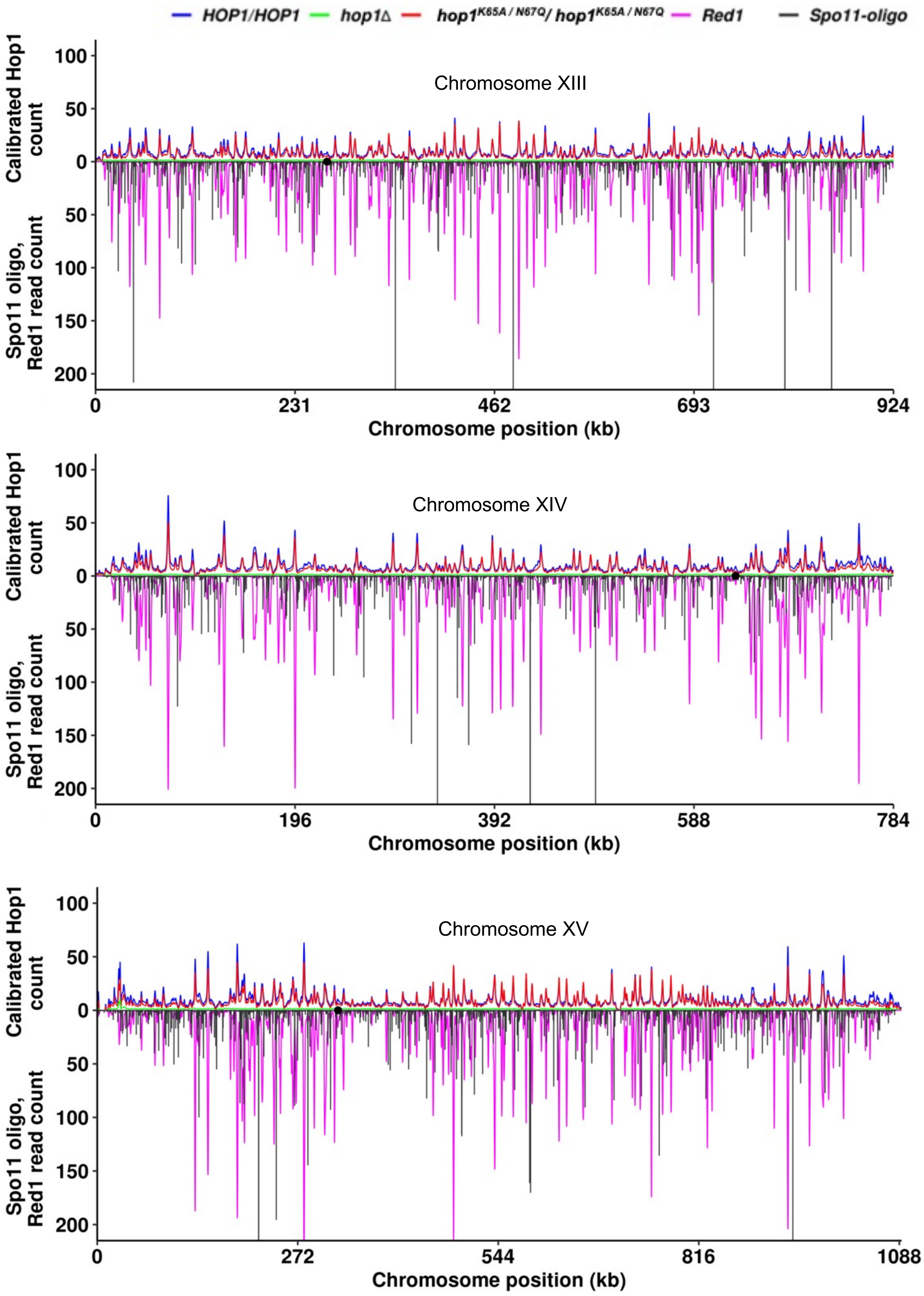
HOP1/HOP1
hop1Δ
hop1K65A / N67Q/ hop1K65A / N67Q
Red1
Spo11-oligo
Chromosome XIII
Chromosome XIV
Chromosome XV
Calibrated Hop1 count
Spo11 oligo, Red1 read count
Chromosome position (kb)

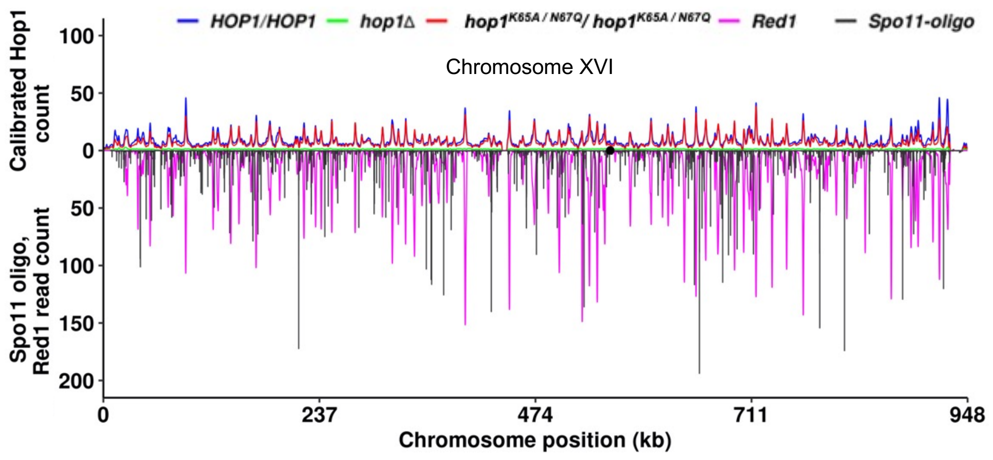

Figure S9. Hop1 occupancy in the *S. cerevisiae* chromosomes of WT, *hop1-K65A,N67Q/hop1-K65A,N67Q* and *hop1Δ/hop1Δ* strains at the 4 h after induction of meiosis. Peaks colored blue, green and red represent the binding pattern of Hop1 in WT, *hop1-K65A,N67Q/hop1-K65A,N67Q and hop1Δ/hop1Δ* strains, respectively. The Spo11 (shown in black) and Red1 (shown in magenta) binding data were taken from literature (1, 2). The centromere is indicated with a black circle. Note: Binding plot for Chromosome III was used for representation in Figure 10A.

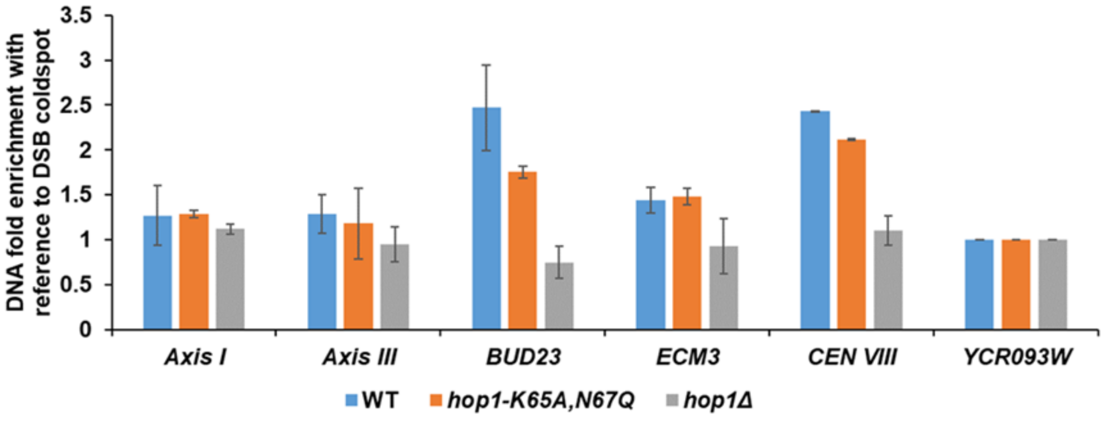

Figure S10. ChIP-qPCR analysis of Hop1 bound to the meiotic chromosomes at the indicated genomic sites. Hop1 enrichment was measured using ChIP qPCR from two independent biological replicates of wild type, *hop1-K65A, N67Q* and *hop1Δ* at DSB hotspots (*BUD23*, *ECM3*), axes (*Axis I*, *Axis III*) and centromere (*CEN VIII*) relative to DSB cold spot (*YCR093W*). The data are normalized with input and plotted relative to the DSB cold spot value. The error bars represent the standard deviation from two independent biological replicates.

References

1. Pan J., Sasaki M., Kniewel R., Murakami H., Blitzblau H.G., Tischfield S.E., Zhu X., Neale M.J., Jasin M., Socci N. D. *et al.* (2011) A hierarchical combination of factors shapes the genome-wide topography of yeast meiotic recombination initiation. *Cell*, **144**, 719-731.

2. Sun X., Huang L., Markowitz T.E., Blitzblau H.G., Chen D., Klein F. and Hochwagen A. (2015) Transcription dynamically patterns the meiotic chromosome-axis interface. *Elife*, **4**, e07424.
